# Comprehensive analyses unveil novel genomic and immunological characteristics of micropapillary pattern in lung adenocarcinoma

**DOI:** 10.3389/fonc.2022.931209

**Published:** 2022-08-03

**Authors:** Yansong Huo, Leina Sun, Jie Yuan, Hua Zhang, Zhenfa Zhang, Lianmin Zhang, Wuhao Huang, Xiaoyan Sun, Zhe Tang, Yingnan Feng, Huilan Mo, Zuoquan Yang, Chao Zhang, Zicheng Yu, Dongsheng Yue, Bin Zhang, Changli Wang

**Affiliations:** ^1^ Department of Lung Cancer, Tianjin Lung Cancer Center, National Clinical Research Center for Cancer, Key Laboratory of Cancer Prevention and Therapy, Tianjin’s Clinical Research Center for Cancer, Tianjin Medical University Cancer Institute and Hospital, Tianjin, China; ^2^ Department of Pathology, National Clinical Research Center for Cancer, Key Laboratory of Cancer Prevention and Therapy, Tianjin’s Clinical Research Center for Cancer, Tianjin Medical University Cancer Institute and Hospital, Tianjin, China; ^3^ GenePlus-Shenzhen, Shenzhen, China

**Keywords:** lung adenocarcinoma, histological subtypes, whole-exome sequencing, copy number alternation, intratumor heterogeneity

## Abstract

Lung adenocarcinoma (LUAD) usually contains heterogeneous histological subtypes, among which the micropapillary (MIP) subtype was associated with poor prognosis while the lepidic (LEP) subtype possessed the most favorable outcome. However, the genomic features of the MIP subtype responsible for its malignant behaviors are substantially unknown. In this study, eight FFPE samples from LUAD patients were micro-dissected to isolate MIP and LEP components, then sequenced by whole-exome sequencing. More comprehensive analyses involving our samples and public validation cohorts on the two subtypes were performed to better decipher the key biological and evolutionary mechanisms. As expected, the LEP and MIP subtypes exhibited the largest disease-free survival (DFS) differences in our patients. *EGFR* was found with the highest mutation frequency. Additionally, shared mutations were observed between paired LEP and MIP components from single patients, and recurrent mutations were verified in the Lung-Broad, Lung-OncoSG, and TCGA-LUAD cohorts. Distinct biological processes or pathways were involved in the evolution of the two components. Besides, analyses of copy number variation (CNV) and intratumor heterogeneity (ITH) further discovered the possible immunosurveillance escape, the discrepancy between mutation and CNV level, ITH, and the pervasive DNA damage response and WNT pathway gene alternations in the MIP component. Phylogenetic analysis of five pairs of LEP and MIP components further confirmed the presence of ancestral *EGFR* mutations. Through comprehensive analyses combining our samples and public cohorts, *PTP4A3*, *NAPRT*, and *RECQL4* were identified to be co-amplified. Multi-omics data also demonstrated the immunosuppression prevalence in the MIP component. Our results uncovered the evolutionary pattern of the concomitant LEP and MIP components from the same patient that they were derived from the same initiation cells and the pathway-specific mutations acquired after *EGFR* clonal mutation could shape the subtype-specificity. We also confirmed the immunosuppression prevalence in the MIP subtype by multi-omics data analyses, which may have resulted in its unfavorable prognosis.

## Introduction

Lung adenocarcinoma (LUAD) is the most common histological type of non-small-cell lung cancer (NSCLC) (1). Most cases of adenocarcinoma are composed of heterogeneous histological subtypes rather than a single one. In the year of 2015, the World Health Organization (WHO) proposed a novel definition of five LUAD subtypes to address the histologic heterogeneity, including the lepidic (LEP), acinar (ACI), papillary (PAP), micropapillary (MIP), and solid (SOL) pattern types. Patients presenting with MIP are prone to lymphovascular invasion and pleural invasion, as well as lymph node or intrapulmonary metastasis after surgical resection (2). Meanwhile, previous studies indicated that patients with a LEP growth pattern exhibited less aggressive behavior and had the most favorable outcomes among the predefined subtypes (3).

Aiming for the elucidation of the mechanisms beyond tumorigenesis and malignance discrepancy, several studies were conducted to evaluate the molecular and genetic features of LUAD subtypes, especially on MIP and LEP. As for the MIP subtype, a recent study observed the disruption of the catenin–cadherin complex (4), which possibly contributed to its poor intercellular adherence. At the genetic level, MIP/SOL tumors have a significantly higher tumor mutation burden (TMB) and fraction of the genome altered than other LUAD subtypes. Key oncogenes *BRAF* and *EGFR* were found with higher mutation frequency in LUAD with MIP in multiregional and multiracial cohorts (5). The gene and protein levels of *c-MET* were also found to be elevated in MIP and patients with a poor prognosis (5, 6). Although dysregulated oncogenes associated with poorer prognoses of MIP-predominant LUAD were identified, there remain key mechanisms that are uncharacterized. For example, the genetic association between subtypes and the evolutional trajectory of the relatively malignant MIP subtype was scarcely discussed.

Noticing the recent emergence of lung cancer immunotherapy, studies assessing the efficacy of immune-related therapies on MIP-predominant LUAD have emerged. Considering the abundance of programmed death-ligand 1 (PD-L1) and programmed cell death protein 1 (PD-1) as well as the tumor immunological microenvironment crucially influence the immunotherapy effectiveness, Francois et al. found the significant differences in PD-L1 expression levels between LUAD histological patterns (7), while Zhang et al. detected higher CD4+ and CD8+ T-cell infiltration as well as increased PD-L1 abundance in samples with a higher percentage of MIP components through the immunohistochemistry staining (8). Regarding the fact that both the studies focused on restricted components of the tumor microenvironment (TME), a more comprehensive analysis of the variation of TME in specific LUAD subtypes could fill the gap in optimal treatment determination, especially for MIP patients.

To address the abovementioned limitations, we retrospectively reviewed 286 patients with different histological subtype-predominance and compared their survival differences. Patients simultaneously possessing MIP and LEP components were further selected for whole-exome sequencing (WES) on both LEP and MIP components, and the genetic differences responsible for variated prognosis and the subtype-level genetic association was investigated. Multi-cohort analyses further discovered the genes specifically altered in MIP or LEP as well as the extent of immune infiltration. Our results expanded the evolution of cognition between the LUAD subtypes and offered therapeutic suggestions for MIP patients.

## Materials and methods

### Patient selection and histopathologic subtyping

We retrospectively reviewed patients diagnosed with LUAD at the Tianjin Cancer Hospital from 2011 to 2014. Among patients who underwent tumor resection, those with an MIP component exceeding 5% of the area size were primarily selected. Patients receiving pre-surgery anticancer treatment, with stage IV disease or other malignancies were excluded. A total of 286 patients passed the selection criteria, and the resected tumors were restaged according to the eighth edition of the American Joint Committee on Cancer TNM staging system for lung cancer. For the LUAD histological subtyping, the formalin-fixed paraffin-embedded (FFPE) samples were first stained with hematoxylin and eosin (H&E) and reviewed by two pathologists. The percentage of each histological component was further calculated in 5% increments and the most dominant pattern was recorded. This study was approved by our institutional review board. Written informed consent was obtained from all patients.

### Sample laser-capture micro-dissection and high-throughput sequencing data generation

Eight FFPE samples from LUAD patients who underwent surgery at the Tianjin Cancer Hospital between 2018 and 2020 were micro-dissected to isolate MIP and LEP components using a NIKON ECLIPSE TI2, Japan microscope. More specifically, 20 FFPE slides were cut into 10 um thick sections, baked for 1 h at 60 °C, stained with H&E, and immersed in xylene. The MIP and LEP areas were circumscribed electronically under the microscope and collected in a centrifugation tube with an adhesive-cap after ablating with a cold laser. Later, genomic DNA of the five pairs of MIP and LEP components plus one MIP component passing initial quality control was extracted by a Maxwell 16 FFPE Plus LEV DNA purification kit and fragmented by an ultra-sonicator UCD-200 (Diagenode, Seraing, Belgium) with length-based selection through Hieff NGS DNA selection beads. DNA quantity was assessed by a Qubit 2.0 Fluorometer with a Quanti-IT dsDNA HS Assay Kit (Thermo Fisher Scientific, MA, USA). The sequencing libraries were further constructed by a custom 53 M whole-exon capturing probe (IDT, IA, USA). The Geneplus-2000 sequencing platform (Geneplus, Beijing, China) further sequenced the libraries in a 100 bp paired-end manner.

### Mutation calling, somatic copy number alteration detection and mutational signature analysis

Raw sequencing data were primarily filtered on the total read volume, GC content, Q30 percentage, and duplication rate. Later read alignment to the human genome (hg19) was performed by BWA (9) (version 0.7.10). After sample coverage filtration, MuTect (10) (version 1.1.4) from the GATK (version 4.0) pipeline identified single nucleotide variants (SNVs), small insertions and deletions (InDels), while segment-level somatic copy number alternations (SCNAs) were detected by GATK. Several rounds of filtration on SNVs were conducted, including (1) retaining variants with low frequency (≤0.01) in a population from the 1,000 Genomes Project (https://www.internationalgenome.org/), the Genome Aggregation Database (gnomAD) (https://gnomad.broadinstitute.org), and the Exome Aggregation Consortium (ExAC); (2) keeping mutations with a number of supporting reads greater than 3; (3) keeping mutations with non-zero variant allele frequency (VAF) (≥0.01); and (4) only functional alternations were preserved. Cancer-associated genes and cancer driver genes were collected from the Cancer Gene Census in the COSMIC database (https://cancer.sanger.ac.uk/census) and two pan-cancer publications (11, 12) for further comparisons. As for SCNAs, an in-house script (13) employed statistical significance between tumor and normal tissues for focal level SCNA inference. Additionally, the mutational spectrum and absolute contribution of COSMIC v3 SBS (single base substitution) mutational signatures were derived by MutationalPatterns (14) on unfiltered somatic mutations, while Sigminer (15) quantified the absolute exposures of COSMIC v3 DBS (double base substitution) and ID (InDel) signatures.

### Intratumor heterogeneity measurement and SNV/SCNA clonal architecture inference

We measured the intratumor heterogeneity (ITH) of samples on both SNV and SCNA. For filtered SNVs, the mutant-allele tumor heterogeneity (MATH) score (16) was calculated using VAF values. The ABSOLUTE (17) tool further estimated the cancer ploidy, tumor purity, rescaled copy ratio, and cancer cell fraction (CCF) by combining SNV and SCNA data. The clonal architectures of SNVs were derived from the higher clonal mutation probability and the CCF upper 95% confidence interval greater than 1. For SCNAs, copy-neutral LOH (CNLOH) segments were initially discarded, and clonal architectures were further annotated using allelic subclonal information from ABSOLUTE outputs. Additionally, by using an in-house script, we constructed the phylogenetic trees by identifying shared and private mutations or focal SCNAs in concomitant MIP and LEP components from one patient. Clonal mutations existing in both components constituted the trunk of the phylogenetic tree, while private mutations constituted the tree branches. Focal SCNA information was also considered in the tree construction procedure, i.e., marking the shared focal SCNA between MIP and LEP.

### Pathway annotation and Gene Oncology analysis

For the integrative analysis of SNVs and SCNAs, DNA damage repair (DDR) related genes were collected from a previous publication (18). The populational structures of mutations were identified on filtered SNVs and annotated SCNAs by PyClone-VI (19). These clone clusters were visualized by ClonEvol (20). The Enrichr (21) tool was used for pathway enrichment and Gene Oncology (GO) analysis. Enriched GO Biological Processes and Reactome (22) pathway entries were reported with P-values.

### Public data curation for comparisons

For multi-omics data comparison, SNV, SCNA, transcriptomic, and proteomic data were retrieved from multiple LUAD datasets. More specifically, SNV and SCNA data from four datasets (Lung-Broad (23), Lung-MSKCC, Lung-OncoSG (24), and TCGA-LUAD) were downloaded from the cBioPortal for Cancer Genomics database (25) or the UCSC Xena database (26) and only non-synonymous mutations were retained. Survival information is also downloaded if available. Additionally, transcriptomic data from the Lung-OncoSG, TCGA-LUAD, and one GEO dataset GSE148801 (27) containing good-prognosis (e.g., LEP, ACI, and PAP histological subtypes) and poor-prognosis (e.g., MIP and SOL) samples were collected while proteomics data from the TCGA-LUAD were similarly curated. Only data from LEP and MIP subtypes were used for further comparisons.

### Immune infiltration analysis by measuring the activity of cancer immunity cycle

Immune infiltration analysis was conducted on curated transcriptomic samples for comparison between LEP and MIP components. Regarding the recognition, response, and killing of cancer cells by the immune system in a step-wise manner, i.e., the cancer immunity cycle (28), we applied the single sample Gene Set Enrichment Analysis (ssGSEA) method from the GSVA R package (http://bioconductor.org/packages/release/bioc/html/GSVA.html) to assess the activities of these steps in the cycle. More specifically, gene signature sets for steps of the cancer immunity cycle were first downloaded from the TIP database (http://biocc.hrbmu.edu.cn/TIP/). Later, the gsva function in the GSVA R package was applied in the step activity quantification procedure.

### Statistical methods

A two-sided Mann–Whitney test was used for evaluating group-level differences between LEP and MIP components. As for multiple comparisons, P-values were adjusted by the Benjamini–Hochberg method. When the comparisons were conducted on categorical data, Fisher’s exact test was used. As for the protein expression data, a one-sided Student’s t-test was used for comparison. For all tests, a P-value (adjusted P-value) <0.05 was considered statistically significant. The Kaplan–Meier (K-M) survival curves were generated by the survminer package (https://rpkgs.datanovia.com/survminer/) and the P-values were calculated using the log-rank test.

## Results

### Clinicopathologic characteristics of selected patients

Among the 286 patients, 51 (17.8%) were LEP-predominant, 178 (62.2%) were ACI-predominant, 16 (5.6%) were PAP-predominant, 29 (10.1%) were MIP-predominant, and 12 (4.2%) were SOL-predominant adenocarcinoma. The clinicopathologic characteristics of all patients are summarized in **Supplementary Table 1**. As shown in **Supplementary Table 2** and **Supplementary Figure 1**, patients with the MIP-predominant subtype exhibited a significantly worse DFS than those with other adenocarcinoma subtypes (P <0.05, **Supplementary Figure 1A**), and LEP and MIP subtypes exhibited the largest DFS difference, while SOL-predominant patients showed significantly worse OS (P <0.05, **Supplementary Figure 1B**).

### Mutational landscape exhibits the involvement of distinct biological processes in LEP and MIP lesions

**Figure 1:**
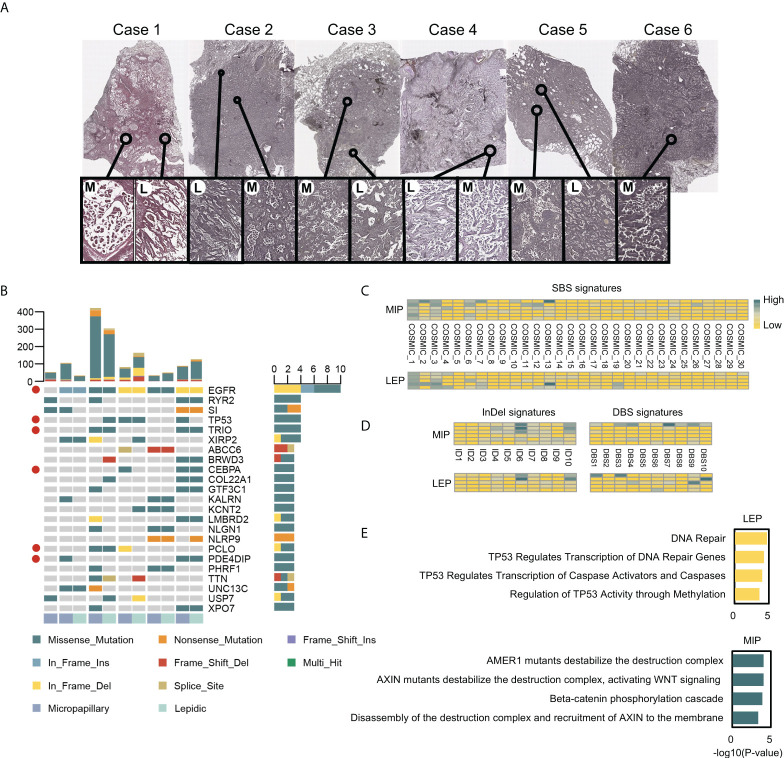
The H&E stained slides and the mutational landscape of MIP and LEP subtypes. **(A)** Scanner view (magnification ×20) of the H&E-stained MIP (M) and LEP (L) subtypes from six patients. **(B)** Genes with top mutation frequency in samples. Cancer-associated genes were marked with red circles. **(C)** Quantifications of the SBS signatures in two histological subtypes. **(D)** Quantifications on the InDel and DBS signatures in subtypes. **(E)** Pathways enriched in mutated tumor suppressor genes (TSGs) for LEP and MIP subtypes.

Among eight micro-dissected samples, six cases passed quality control, but the quantity of LEP component in one case was inadequate. Six MIP and five LEP components were finally sequenced (**Supplementary Table 3** and **Figure 1A**). A total of 2,035 and 2,757 SNVs and InDels were identified, while 684/791 and 257/284 mutations were retained after quality and cancer-related gene filtration (**Supplementary Figure 2A**and **Supplementary Table 4**). Genes with the highest mutation frequency after quality filtering are shown in **Figure 1B**. *EGFR* was identified to be the most frequently mutated drive gene, in line with the finding that LEP and MIP components possess significantly higher *EGFR* mutation frequencies (29). Besides, several cancer-associated genes including *TP53*, *TRIO*, *CEBPA*, *PCLO*, and *PDE4DIP* were concomitantly mutated (**Figure 1B**), denoting *p53*, WNT-beta-catenin signaling, *PI3K*/*AKT*/*mTOR* signaling, and DNA repair pathways were affected. Interestingly, shared mutations were observed between paired LEP and MIP components from single patients (**Figure 1B**), raising the presumption that the paired LEP and MIP components could be homogeneous. We also compared the mutation frequency of these genes with public cohorts, including Lung-Broad, Lung-OncoSG, and TCGA-LUAD. Several cancer-associated genes, including *EGFR*, *TP53*, *TRIO*, *PCLO*, and *PDE4DIP*, were found recurrently mutated (**Supplementary Figure 2B**).

Mutation signature analysis was conducted on unfiltered mutations separately for LEP and MIP components. The point substitution spectrum plot displayed an insignificant difference between the two histological subtypes (**Supplementary Figure 2C**). Similarly, the SBS, InDel, and DBS signatures mapped to the COSMIC database (accessed in March 2021) were similar between the two subtypes (**Figures 1C, D**), indicating the histological differences between LEP and MIP components could be caused by alternations in specific key genes.

Of particular interest, mutated tumor suppressor genes (TSGs) were enriched in distinct pathways in the LEP and MIP subtypes (**Figure 1E**). TSGs from LEP components were enriched in DNA repair and *TP53*-related pathways, while mutated TSGs in MIP components were found to be enriched in pathways associated with beta-catenin destruction complex, *AXIN* mutation and WNT signaling, which followed report (4). When concerning the enriched pathways for mutated oncogenes, seven of the 10 top-enriched Reactome pathways from the two groups were identical, which were mainly associated with *EGFR* and *PI3K* signaling (**Supplementary Figure 2D**). By inspecting the mutated TSG pathway enrichment pattern in Lung-Broad (**Supplementary Figures 3A, B**), Lung-OncoSG (**Supplementary Figures 3C, D**), and TCGA-LUAD (**Supplementary Figures 3E, F**) cohorts, similar entries were identified in both LEP and MIP samples, while *NOTCH1*-related pathways were additionally found in TCGA-LUAD MIP samples, which was unsurprising since the cross-talk between NOTCH and WNT pathways was previously unveiled (30). As for the oncogenes mutated in three public cohorts, shared terms were found between LEP and MIP components in Lung-Broad (**Supplementary Figures 4A, B**), Lung-OncoSG (**Supplementary Figures 4C, D**), and TCGA-LUAD (**Supplementary Figures 4E, F**) cohorts but with lower overlapping proportion, which endorsed the possible existence of a common mutational ancestor in the paired components.

### Copy number alternation and clonality analysis uncovered distinct ITH characteristics in LEP and MIP subtypes

**Figure 2:**
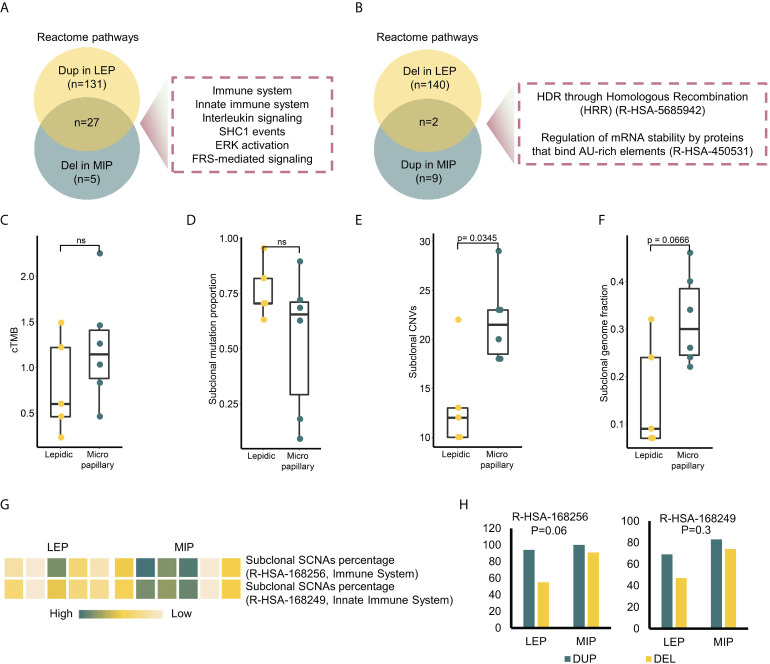
Multi-perspective investigation on intratumor heterogeneity (ITH) difference between two subtypes. **(A)** Intersection of the enriched pathways in genes uniquely amplified in LEP and deleted in MIP. **(B)** Intersection of the enriched pathways in genes uniquely deleted in LEP and amplified in MIP. **(C–F)** Clonal tumor mutation burden (cTMB), subclonal mutation proportion, subclonal CNV and subclonal genome fraction distribution in two subtypes. **(G)** Subclonal SCNA percentage of two Reactome immune pathways in sequenced samples. **(H)** Focal alternation number on the genes in two immune pathways. P-values on the alternation discrepancy between subtypes were calculated by Fisher’s exact test.

Through the segment-level copy number alternation identification procedures, multiple amplified and deleted segments were detected (**Supplementary Figure 5A**). Chromosomes including 3,4,5,10,15,17, and 18 exhibited different copy number alternation patterns between the two subtypes, and the MIP subtype showed both higher chromosome level (**Supplementary Figure 5B**) and arm-level copy number variation (CNV) burden (**Supplementary Figure 5C**), which followed the report (31). To further pinpoint the recurrent SCNAs at the focal level, we identified 1,116 genes with somatic copy number alternations through statistical testing on read coverages from all samples (details in *Materials and methods*), among which 159/80 genes were uniquely amplified/deleted in the LEP component, while 34/11 genes were uniquely amplified/deleted in the MIP component. By annotating the enriched pathways on these genes, 27 pathways were found to overlap between the enrichment results of uniquely amplified genes in LEP and deleted genes in MIP, which could be categorized into the immune system, innate immune system, interleukin signaling, *SHC1* events, *ERK* activation, and *FRS*-mediated signaling pathways (**Figure 2A**). When inspecting the number of genes, pathways related to the immune system and innate immune system got the highest gene number variated (37 genes amplified in LEP and four genes deleted in MIP subtype), indicating that MIP LUADs tend to have induced immunosurveillance escape. Additionally, two pathways were identical between the enrichment results of uniquely deleted genes in LEP and amplified genes in MIP (**Figure 2B**), which were associated with homology directed repair (HDR) and mRNA fate regulation, but the variated gene number was limited (six genes for LEP and four genes for the MIP group).

Intratumor heterogeneity (ITH) can depict the genetic and epigenetic tumor inner diversity and is proven to be closely related to cancer progression, therapeutic resistance, and recurrences. To compare the ITH of the two histological subtypes at both the mutational and copy number level, we annotated the mutations/SCNAs with clonality. As shown in **Figure 2C**, there was no significant difference in the clonal tumor mutation burden (cTMB) and subclonal mutation proportion between the MIP and LEP groups (**Figure 2D**). The MATH score, which is widely used to measure the mutational ITH, exhibited a similar trend (**Supplementary Figure 5D**). As for the copy number variations, the MIP group possessed a significantly higher proportion of subclonal SCNAs (**Figure 2E**) as well as a trend of higher subclonal genome fraction (**Figure 2F**). Interestingly, the frequency of clonal mutations in DNA Damage Response (DDR) and WNT pathway genes was higher in the MIP subtype (**Supplementary Figure 5E**), which may possibly partially increase the subclonal genome alternations in immune-related genes since the association between canonical WNT-beta-catenin signaling and carcinogenesis as well as immune suppression was clear (32). Indeed, six MIP components showed a trend of a higher percentage of subclonal SCNA (**Figure 2G**) as well as a higher number of focal deletions (**Figure 2H**) on the genes related to the two immune pathways. We confirmed the results that genes significantly deleted in the MIP subgroup exhibited association with immune-related terms in the Lung-Broad and Lung-OncoSG cohorts (**Supplementary Figure 6**).

### Evolutionary pattern exploration on the paired LEP and MIP components

**Figure 3:**
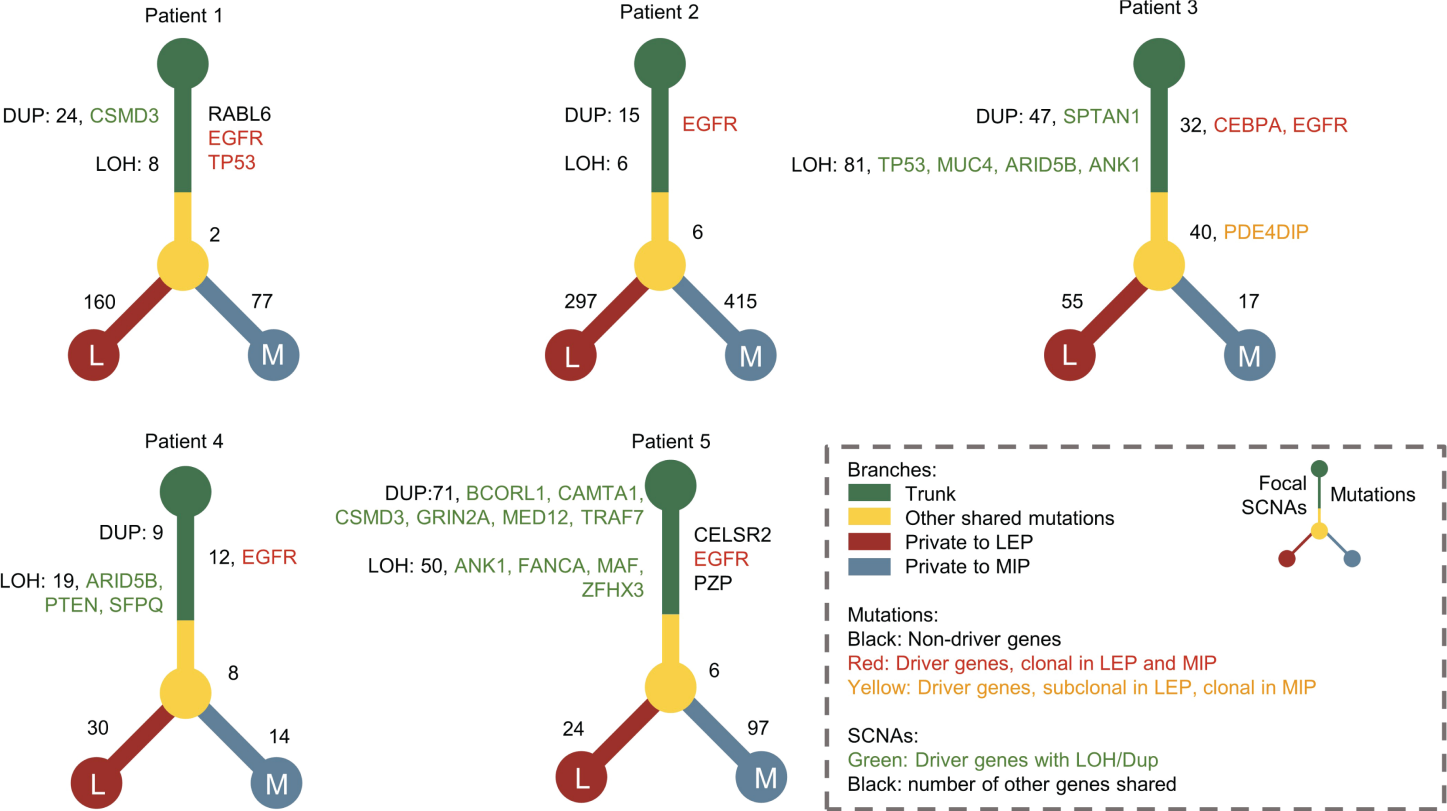
The phylogenetic trees constructed for patients with concomitant MIP and LEP components. Driver genes with mutations and focal CNV were marked with different colors. The numbers of shared mutations and focal CNVs were labeled beside the tree trunk, while mutation numbers private to MIP and LEP components were labeled beside the tree branches.

To elaborate on the possible evolutionary process between LEP and MIP subtypes, we delineated the phylogenetic trees for each patient based on mutations as well as focal level SCNAs. As shown in **Figure 3**, all five patients possessed truncal mutations between paired LEP and MIP components, while no obvious bias on private mutation burden after truncal divergence was observed. Clonal mutations on cancer drivers including *EGFR*, *TP53*, and *CEBPA* were identified, and *EGFR* was the only gene coincident in five pairs, which confirmed the presence of ancestral mutations. The driver mutations private to LEP were enriched in chromatin organization, *TP53*-related and DNA double strand repair pathways (**Supplementary Figure 7A**), while mutations private to MIP were enriched in cellular signaling and beta-catenin-related pathways (**Supplementary Figure 7B**). We further annotated the shared mutations in **Figure 3** with clonality to explore the clonal-subclonal transitions between the LEP and MIP subtypes. For the genes possessing mutations with increased clonality in MIP, GO terms related to neurogenesis were found enriched (**Supplementary Figure 7C**), denoting that tumor-induced neurogenesis and nerve-cancer crosstalk may account for the aggressiveness of the MIP subtype. Oppositely, genes with mutations switched from subclonal to clonal in LEP were associated with cell-cycle related GO biological processes (**Supplementary Figure 7D**). As for the truncal focal SCNAs, several driver genes including *CSMD3*, *SPTAN1*, *BCORL1*, *CAMTA1*, *GRIN2A*, *MED12*, and *TRAF7* were concurrently amplified in the two subtypes (**Figure 3**), which were associated with developmental biology (R-HSA-1266738) and *EGFR*-related Reactome pathways (R-HSA-179812 and R-HSA-180336). Moreover, the deletion of *TP53*, *MUC4*, *ARID5B*, *ANK1*, *PTEN*, *SFPQ*, *FANCA*, *MAF*, and *ZFHX3* were observed in the two subtypes. Interestingly, no driver gene showed concordant copy number variation in five pairs of samples, possibly due to the elevated SCNA-level ITH in the MIP group. We also scrutinized the genes with shared copy number variation in the paired samples. As shown in **Supplementary Figure 8A**, most shared deletions were on immune-related genes, while signal transduction and *PI3K*/*AKT* pathways, which abnormality is highly associated with tumor progression and therapeutic resistance, were found uniformly amplified (**Supplementary Figure 8B**). To further derive the mutational transitions and evolutionary trajectory, we used PyClone-VI to infer the mutational populations and their evolution among paired components. As shown in **Supplementary Figure 9A**, numerous clone clusters were identified in 5 patients, which exhibited dynamic variant allele frequency (VAF) alternation. Clusters with drastically increased VAF in the LEP subtype were mainly enriched in mRNA splicing pathways (**Supplementary Figure 9B**), while clusters with increased VAF in the MIP subtype were associated with *ERBB2* functions (**Supplementary Figure 9C**). These data imply that LEP and MIP components from one patient were derived from the same initiation cells and the pathway-specific mutations acquired after the *EGFR* clonal mutation eventually shaped the subtype-specificity.

### Group-wise comparison discovered co-amplified pattern of PTP4A3, NAPRT, and RECQL4

We next gathered SNV and SCNA data and identified the genes with an alternation frequency difference between the LEP and MIP groups. As shown in **Table 1**, mutation frequency difference was observed on nine genes, with three genes specifically mutated in LEP group. Additionally, five genes were found with a distinct copy number alternation pattern, with one gene specifically amplified in the LEP group (**Table 1**). Similarly, mutation frequencies on the above nine genes and the key gene identified in phylogenetic analyses (*EGFR*) were inspected in four public cohorts (**Table 2**), and *EGFR* was the only gene with a significant mutational difference in non-east Asian public cohorts. Moreover, the five SCNA genes showed an alternation frequency difference in three non-east Asian cohorts (**Table 2**). Interestingly, the altered sample proportion or the alternation frequency for *PTP4A3*, *NAPRT*, and *RECQL4* was highly similar between our MIP group (**Table 1**) and public cohorts (Lung-Broad, Lung-OncoSG, and TCGA-LUAD, **Table 2**), implying the feasibility of their cooperative function through duplication in MIP components. Spearman’s correlation coefficient (SCC) on the SCNA pattern of our 11 samples confirmed the association of the co-amplified genes (**Supplementary Figure10 A**). Such strong association was also observed on SCNA data for LEP and MIP adenocarcinoma from Lung-Broad (**Supplementary Figure 10B**), Lung-OncoSG (**Supplementary Figure 10C**, left), and TCGA-LUAD (**Supplementary Figure 10D**, left) cohorts. Concerning the fact that SCNA is highly related to the consequent gene expression alternation, we calculated the expressional SCC of the five genes in cohorts with available transcriptomic data. When compared to all samples (**Supplementary Figures 10C, D**, middle), the expressional associations between *PTP4A3*, *NAPRT*, and *RECQL4* transformed to a higher synergetic state for LEP and MIP samples in both the Lung-OncoSG (**Supplementary Figure 10C**, right) and TCGA-LUAD (**Supplementary Figure 10D**, right) cohorts. More explicitly, the correlation between SCNA and RNA expression was higher for the three genes in two public cohorts (**Supplementary Figure 10E**), and *NAPRT* as well as *PTP4A3* exhibited significantly higher LEP/MIP group-specificity. As an exemplification, the correlation between the SCNA and RNA expression of the *PTP4A3* gene increased from 0.372 to 0.693 when narrowed to only LEP/MIP samples in the TCGA-LUAD cohort (**Supplementary Figures 10F, G**). All three co-amplified genes were significantly overexpressed in tumor samples (**Supplementary Figures 10H–J**, left) and *RECQL4* possessed significantly higher expression levels in the MIP subtype (**Supplementary Figure 10J**, right) in the TCGA-LUAD cohort. To conclude, our comprehensive analyses identified *PTP4A3*, *NAPRT*, and *RECQL4* were co-amplified and co-expressed specifically in LEP/MIP adenocarcinoma, and *RECQL4* was upregulated in the MIP group.

**Table 1:** Genes exhibited subtype-specific alternation frequency among the sequenced samples.

Genes	Number of samples altered in each subtype		Number of mutations in each subtype		Alternation Type
	LEP (a total of 5)	MIP (total 6)	LEP (a total of 5)	MIP (a total of 6)	
C10orf71	1	0	3	0	Mutation
SLC32A1	1	0	4	0	Mutation
DISC1	1	0	5	0	Mutation
AHCTF1	0	1	0	3	Mutation
PHRF1	1	2	1	3	Mutation
PLEC	0	1	0	3	Mutation
RYR2	1	3	1	3	Mutation
SI	1	3	0	3	Mutation
SYNE2	1	2	1	4	Mutation
RCSD1	3	0			Duplication
PTP4A3	1	5			Duplication
EZR	2	5			Deletion
NAPRT	2	5			Duplication
RECQL4	2	5			Duplication

**Table 2:** Inspection on the alternation frequency of genes exhibited subtype-specific alternations using four public cohorts.

Genes	Number of samples altered in each subtype												Alternation Type
	Lung-Broad			Lung-MSKCC			Lung-OncoSG			TCGA-LUAD			
	LEP (a total of 13)	MIP (a total of 17)	Adj. P-value	LEP (a total of 88)	MIP or Solid (a total of 105)	P-value	LEP (a total of 10)	MIP (a total of 4)	Adj. P-value	LEP (a total of 12)	MIP (a total of 23)	Adj. P-value	
EGFR	4	2	0.00052	38	19	7.3E−05	6	3	0.525	2	4	0.00246	Mutation
SLC32A1	0	1	0.626							1	1	1	Mutation
DISC1										0	1	1	Mutation
AHCTF1	0	1	0.626							2	1	1	Mutation
PHRF1	0	1	0.13904							0	1	0.34034	Mutation
PLEC	0	3	1							1	2	1	Mutation
RYR2	1	7	0.80126				2	1	0.656	2	7	1	Mutation
SI	1	5	0.626				2	0	0.525	1	4	1	Mutation
SYNE2	0	1	0.13904							1	4	1	Mutation
RCSD1	4	4	1				6	3	0.5535	10	20	0.00278	Duplication
PTP4A3	3	1	0.0502				5	2	1	6	10	1	Duplication
EZR	0	1	0.00042				2	2	0.58	8	13	1	Deletion
NAPRT	3	1	0.01233				5	2	1	6	10	1	Duplication
RECQL4				2	1	1.4E−08	5	2	1	6	10	1	Duplication

### Immune-related analyses uncovered elevated immunosuppression in MIP subtype

The disparity of cancer immunity cycle activity was examined in the Lung-OncoSG, GEO, and TCGA-LUAD cohorts as described in *Materials and methods*. Activities of recurrent cancer immunity steps including the release of cancer cell antigen, CD8+ T-cell recruiting, dendritic cell recruiting, macrophage recruiting, T-helper 17 (Th17) cell recruiting, T-cell infiltration into tumors and killing of cancer cells were significantly higher in MIP subtype (**Supplementary Figures 11A–C**). By further examining the differentially expressed proteins between LEP and MIP subtypes from the TCGA-LUAD dataset, the identified proteins with MIP-specific elevation (**Supplementary Figure 12A**) were significantly enriched in *PD-L1* and *PD-1* checkpoint pathways in cancer (**Supplementary Figure 12B**). These data suggest that the MIP subtype could exist in an immune-suppressive microenvironment.

## Discussion

Consistent with other studies, we confirmed the survival disparity between LUAD subtypes. By performing WES on micro-dissected LUAD tissue samples of MIP and LEP components, we explored the genetic features related to the LEP/MIP growth pattern and the evolutional connection between LUAD subtypes. Our results revealed that LEP and MIP subtypes could be derived from the same initiation cells with *EGFR* mutation and the ultimate histological dissimilitude was shaped by the pathway-specific mutations acquired along evolution. Our results showed that the *EGFR* trunk mutation arose between pre-invasive and invasive LUAD and LEP/MIP components were evolved by a branched evolution model.

Through comprehensive comparisons of genetic alternations, the biological characteristics of the two LUAD subtypes were elucidated. As for mutational comparisons, TSG mutations in LEP were associated with DNA repair and *TP53* regulation, while genes related to WNT signaling and beta-catenin destruction complexes got both higher mutational frequency and clonality. Driver mutations private to MIP were also enriched in cellular signaling and beta-catenin-related pathways, while genes that possessed lower mutational heterogeneity in MIP were associated with neurogenesis and *ERBB2* functions. Aberrant WNT signaling pathway activation caused by gene mutations of intracellular components is associated with a higher rate of recurrence in early-stage NSCLC. On the other hand, being the critical downstream effector in the canonical WNT pathway, excessive intracellular beta-catenin promotes lung cancer aggression. Liang et al. further confirmed that the intracellular beta-catenin expression in MIP-predominant LUAD was higher than LEP-predominant LUAD (4). Besides, neurogenesis induced by tumors shapes an immunosuppressive microenvironment (33). Although cancer-related neurogenesis is considered to be associated with solid tumor metastasis, its role in LUAD remains poorly understood. Our results suggest an inner association between the MIP aggressive phenotype and neurogenesis. The activation of well-known proto-oncogene *ERBB2* signaling was associated with poor outcomes in NSCLC (34), coinciding with MIP characteristics. The copy number of genes related to the immune system, innate immune system, interleukin signaling, SHC1 events, ERK activation, and FRS-mediated signaling were also found to decrease in MIP. With the highest proportion of immune genes affected, the immunosuppression status in the MIP subtype was confirmed. Apart from specified genomic alternations, ITH provides crucial information for drug responsiveness and clinical prognosis. Discordance between SNV and SCNA ITH was particularly observed in the MIP subtype. Subclonal genetic instability possibly facilitated MIP neoplastic cell proliferation (35) and the clonal mutations on key MIP-specific pathways contributed to its aggressive behavior.

We discovered three genes with co-amplification tendency, both in our discovery cohort and in three public validation cohorts. Previous studies proved that the knockdown of *PTP4A3* inhibited cell migration and invasion of lung cancer cell lines (36). It also induced microvascular and lymphatic vessel formation by increasing VEGF and VEGF-C expression in lung cancer tissues, which was in accordance with the clinical observations that the MIP component in LUAD increases the risk of distant and lymph node metastasis. A previous study also found that the loss of *NAPRT* promoted the epithelial–mesenchymal transition (EMT) by stabilizing beta-catenin (37). The elevated expression of *NAPRT* was conceivably associated with the disruption of the catenin–cadherin complex in MIP. Moreover, *RECQL4* could coordinate and regulate cell proliferation and cell cycle progression by protecting chromosome stability (38), and its protein expression was remarkably higher in LUAD (39). The biological mechanisms of these three genes further verified our discoveries.

Interestingly, we observed numerous genetic alternations associated with the immune status in the LEP and MIP subtypes. For example, the enrichment of immune-related pathways, including immune system and innate immune system, was observed on genes uniquely amplified in LEP and deleted in the MIP category. Along with the observation of a higher percentage of focal deletions on the immune pathway genes in our cohort and the deficiency in immune-related pathways for MIP group-specific deleted genes in Lung-Broad and Lung-OncoSG cohorts, we suspected that the MIP subtype could possess an immuno-suppression microenvironment, in other words, an induced immuno-surveillance escape. Regarding the increasing enthusiasm for lung cancer immunotherapy and our hypothesis, we next assessed and compared the TME landscape between the MIP and LEP components using the stepwise activities of the cancer immunity cycle. Steps including CD8+ T-cell recruiting, T-helper 17 (Th17) cell recruiting, T-cell infiltration into tumors and killing of cancer cells were significantly higher in MIP samples. However, elevated T-cell infiltration does not always indicate better clinical outcomes for patients. For instance, elevated expression of PD-L1 and PD-1 could inhibit the activation of T cells, conferring an immuno-evasion and immuno-suppression tumor status. Unsurprisingly, proteomic analysis further confirmed the activation of the PD1/PD-L1 pathway in the MIP subtype, partially elucidating the deteriorative survival of MIP patients. Generally, our work revealed the comprehensive TME situation of LEP/MIP components and immuno-suppression features in MIP-predominant LUAD.

Our study has several limitations. Firstly, the cohort only included five pairs of LEP/MIP components detached from five LUAD patients and 11 samples. Further studies with a larger amount of patient involvement can better decipher the evolutionary trajectory between LUAD histological subtypes and identify subtype-specific genetic changes. Moreover, the three genes with co-amplification or co-expression tendency should be further experimentally validated, particularly on their protein expression status. Additionally, we portrayed the TME heterogeneity using bulk RNA-seq data. With the recent maturation of multiple advanced techniques, using methods including single-cell RNA-seq, spatial transcriptomics, and multiplexed immunohistochemistry could better dissect the TME in LUAD. Lastly, our analyses only focused on MIP and LEP subtypes. A more integrated study incorporating other LUAD histologic subtypes could better decode the disease.

To conclude, we identified subtype-specific genetic differences responsible for variated prognosis and the evolution trajectory of the MIP subtype. The subtype-specificity was possibly shaped by pathway-specific mutations acquired after the *EGFR* clonal mutation. The tumor microenvironment revealed the immunosuppression prevalence in MIP, which could contribute to its unfavorable prognosis. Immune checkpoint inhibitor treatments like anti-PD-1/anti-PD-L1 could maximize the therapeutic benefit for MIP-predominant LUAD patients.

## Data availability statement

The raw sequencing datasets presented in this study can be found in the China National GeneBank DataBase (https://db.cngb.org/) with project number CNP0003191.

## Ethics statement

The studies involving human participants were reviewed and approved by the Tianjin Medical University Cancer Institute and Hospital review board. The patients/participants provided their written informed consent to participate in this study.

## Author contributions

CW, BZ, and DY contributed to the study design. LS, ZZ, LZ, YH, WH, XS, ZT, YF, and HM contributed to data collection. JY, HZ, ZuY, CZ, and ZiY performed statistical analysis and data interpretation. YH and JY drafted the manuscript. CW and BZ revised the manuscript. CW and DY provided financial support and study supervision. All authors listed have made a substantial, direct, and intellectual contribution to the work and approved it for publication.

## Funding

This work was financially supported by the National Natural Science Foundation of China (grant number 81772484 to CW, grant number 82173038 to DY).

## Conflict of interest

Authors JY, ZuY, CZ, and ZiY were employed by GenePlus-Shenzen.

The remaining authors declare that the research was conducted in the absence of any commercial or financial relationships that could be construed as a potential conflict of interest.

## Publisher’s note

All claims expressed in this article are solely those of the authors and do not necessarily represent those of their affiliated organizations, or those of the publisher, the editors and the reviewers. Any product that may be evaluated in this article, or claim that may be made by its manufacturer, is not guaranteed or endorsed by the publisher.

## References

[1] Bray F Ferlay J Laversanne M Brewster DH Gombe Mbalawa C Kohler B. Cancer incidence in five continents: Inclusion criteria, highlights from volume X and the global status of cancer registration. Int J Cancer (2015) 137(9):2060–71. doi: 10.1002/ijc.29670 26135522

[2] Hung JJ Yeh YC Jeng WJ Wu YC Chou TY Hsu WH. Factors predicting occult lymph node metastasis in completely resected lung adenocarcinoma of 3 Cm or smaller. Eur J Cardiothorac Surg (2016) 50(2):329–36. doi: 10.1093/ejcts/ezv485 26819290

[3] Makinen JM Laitakari K Johnson S Makitaro R Bloigu R Lappi-Blanco E. Nonpredominant lepidic pattern correlates with better outcome in invasive lung adenocarcinoma. Lung Cancer (2015) 90(3):568–74. doi: 10.1016/j.lungcan.2015.10.014 26506915

[4] Zhu L Yang S Zheng L Zhang G Cheng G. Wnt/Beta-catenin pathway activation *Via* Wnt1 overexpression and Axin1 downregulation correlates with cadherin-catenin complex disruption and increased lymph node involvement in micropapillary-predominant lung adenocarcinoma. J Thorac Dis (2020) 12(10):5906–15. doi: 10.21037/jtd-20-1495 PMC7656375 33209423

[5] Warth A Penzel R Lindenmaier H Brandt R Stenzinger A Herpel E. Egfr, kras, braf and alk gene alterations in lung adenocarcinomas: Patient outcome, interplay with morphology and immunophenotype. Eur Respir J (2014) 43(3):872–83. doi: 10.1183/09031936.00018013 23988776

[6] Tsao MS Marguet S Le Teuff G Lantuejoul S Shepherd FA Seymour L. Subtype classification of lung adenocarcinoma predicts benefit from adjuvant chemotherapy in patients undergoing complete resection. J Clin Oncol (2015) 33(30):3439–46. doi: 10.1200/JCO.2014.58.8335 PMC4606061 25918286

[7] Ng Kee Kwong F Laggner U McKinney O Croud J Rice A Nicholson AG. Expression of pd-L1 correlates with pleomorphic morphology and histological patterns of non-Small-Cell lung carcinomas. Histopathology (2018) 72(6):1024–32. doi: 10.1111/his.13466 29323731

[8] Zhang S Xu Y Zhao P Bao H Wang X Liu R. Integrated analysis of genomic and immunological features in lung adenocarcinoma with micropapillary component. Front Oncol (2021) 11:652193. doi: 10.3389/fonc.2021.652193 34221970 PMC8248503

[9] Li H. Aligning sequence reads, clone sequences and assembly contigs with BWA-MEM. arXiv (2013) 1303.3997. doi: 10.48550/arXiv.1303.3997

[10] Cibulskis K Lawrence MS Carter SL Sivachenko A Jaffe D Sougnez C. Sensitive detection of somatic point mutations in impure and heterogeneous cancer samples. Nature Biotechnology (2013) 31(3):213–9. doi: 10.1038/nbt.2514 PMC3833702 23396013

[11] Bailey MH Tokheim C Porta-Pardo E Sengupta S Bertrand D Weerasinghe A. Comprehensive characterization of cancer driver genes and mutations. Cell (2018) 173(2):371–85. e18. doi: 10.1016/j.cell.2018.02.060 29625053 PMC6029450

[12] Vogelstein B Papadopoulos N Velculescu VE Zhou S Diaz LA Jr. Kinzler KW. Cancer genome landscapes. Science (2013) 339(6127):1546–58. doi: 10.1126/science.1235122 PMC3749880 23539594

[13] McGranahan N Rosenthal R Hiley CT Rowan AJ Watkins TBK Wilson GA. Allele-specific hla loss and immune escape in lung cancer evolution. Cell (2017) 171(6):1259–71.e11. doi: 10.1016/j.cell.2017.10.001 29107330 PMC5720478

[14] Blokzijl F Janssen R van Boxtel R Cuppen E. Mutationalpatterns: Comprehensive genome-wide analysis of mutational processes. Genome Med (2018) 10(1):33. doi: 10.1186/s13073-018-0539-0 29695279 PMC5922316

[15] Wang S Tao Z Wu T Liu XS. Sigflow: An automated and comprehensive pipeline for cancer genome mutational signature analysis. Bioinformatics (2021) 37(11):1590–2. doi: 10.1093/bioinformatics/btaa895 PMC8275980 33270873

[16] Mroz EA Rocco JW. Math, a novel measure of intratumor genetic heterogeneity, is high in poor-outcome classes of head and neck squamous cell carcinoma. Oral Oncol (2013) 49(3):211–5. doi: 10.1016/j.oraloncology.2012.09.007 PMC3570658 23079694

[17] Carter SL Cibulskis K Helman E McKenna A Shen H Zack T. Absolute quantification of somatic DNA alterations in human cancer. Nat Biotechnol (2012) 30(5):413–21. doi: 10.1038/nbt.2203 PMC4383288 22544022

[18] Knijnenburg TA Wang L Zimmermann MT Chambwe N Gao GF Cherniack AD. Genomic and molecular landscape of DNA damage repair deficiency across the cancer genome atlas. Cell Rep (2018) 23(1):239–54 e6. doi: 10.1016/j.celrep.2018.03.076 29617664 PMC5961503

[19] Gillis S Roth A. Pyclone-vi: Scalable inference of clonal population structures using whole genome data. BMC Bioinf (2020) 21(1):571. doi: 10.1186/s12859-020-03919-2 PMC7730797 33302872

[20] Dang HX White BS Foltz SM Miller CA Luo J Fields RC. Clonevol: Clonal ordering and visualization in cancer sequencing. Ann Oncol (2017) 28(12):3076–82. doi: 10.1093/annonc/mdx517 PMC5834020 28950321

[21] Kuleshov MV Jones MR Rouillard AD Fernandez NF Duan Q Wang Z. Enrichr: A comprehensive gene set enrichment analysis web server 2016 update. Nucleic Acids Res (2016) 44(W1):W90–7. doi: 10.1093/nar/gkw377 PMC4987924 27141961

[22] Fabregat A Jupe S Matthews L Sidiropoulos K Gillespie M Garapati P. The reactome pathway knowledgebase. Nucleic Acids Res (2018) 46(D1):D649–55. doi: 10.1093/nar/gkx1132 PMC5753187 29145629

[23] Imielinski M Berger AH Hammerman PS Hernandez B Pugh TJ Hodis E. Mapping the hallmarks of lung adenocarcinoma with massively parallel sequencing. Cell (2012) 150(6):1107–20. doi: 10.1016/j.cell.2012.08.029 PMC3557932 22980975

[24] Chen J Yang H Teo ASM Amer LB Sherbaf FG Tan CQ. Genomic landscape of lung adenocarcinoma in East asians. Nat Genet (2020) 52(2):177–86. doi: 10.1038/s41588-019-0569-6 32015526

[25] Gao J Aksoy BA Dogrusoz U Dresdner G Gross B Sumer SO. Integrative analysis of complex cancer genomics and clinical profiles using the cbioportal. Sci Signaling (2013) 6(269):pl1–pl. doi: 10.1126/scisignal.2004088 PMC4160307 23550210

[26] Goldman M Craft B Hastie M Repečka K McDade F Kamath A. Visualizing and interpreting cancer genomics data via the Xena platform. Nature Biotechnol (2020) 38(6):675–8. 10.1038/s41587-020-0546-8 PMC7386072 32444850

[27] Tone M Tahara S Nojima S Motooka D Okuzaki D Morii E. Htr3a is correlated with unfavorable histology and promotes proliferation through erk phosphorylation in lung adenocarcinoma. Cancer Sci (2020) 111(10):3953–61. doi: 10.1111/cas.14592 PMC7540989 32736413

[28] Chen DS Mellman I. Oncology meets immunology: The cancer-immunity cycle. Immunity (2013) 39(1):1–10. doi: 10.1016/j.immuni.2013.07.012 23890059

[29] Li H Pan Y Li Y Li C Wang R Hu H. Frequency of well-identified oncogenic driver mutations in lung adenocarcinoma of smokers varies with histological subtypes and graduated smoking dose. Lung Cancer (2013) 79(1):8–13. doi: 10.1016/j.lungcan.2012.09.018 23098378

[30] Krishnamurthy N Kurzrock R. Targeting the Wnt/Beta-catenin pathway in cancer: Update on effectors and inhibitors. Cancer Treat Rev (2018) 62:50–60. doi: 10.1016/j.ctrv.2017.11.002 29169144 PMC5745276

[31] Caso R Sanchez-Vega F Tan KS Mastrogiacomo B Zhou J Jones GD. The underlying tumor genomics of predominant histologic subtypes in lung adenocarcinoma. J Thorac Oncol (2020) 15(12):1844–56. doi: 10.1016/j.jtho.2020.08.005 PMC7704768 32791233

[32] Chehrazi-Raffle A Dorff T Pal S Lyou Y. Wnt/B-catenin signaling and immunotherapy resistance: Lessons for the treatment of urothelial carcinoma. Cancers (2021) 13:889. doi: 10.1038/s41392-020-0205-z 33672668 PMC7924395

[33] Cervantes-Villagrana RD Albores-Garcia D Cervantes-Villagrana AR Garcia-Acevez SJ. Tumor-induced neurogenesis and immune evasion as targets of innovative anti-cancer therapies. Signal Transduct Target Ther (2020) 5(1):99. doi: 10.1038/s41392-020-0205-z 32555170 PMC7303203

[34] Riudavets M Sullivan I Abdayem P Planchard D. Targeting Her2 in non-Small-Cell lung cancer (Nsclc): A glimpse of hope? an updated review on therapeutic strategies in nsclc harbouring Her2 alterations. ESMO Open (2021) 6(5):100260. doi: 10.1016/j.esmoop.2021.100260 34479034 PMC8414039

[35] Dentro SC Leshchiner I Haase K Tarabichi M Wintersinger J Deshwar AG. Characterizing genetic intra-tumor heterogeneity across 2,658 human cancer genomes. Cell (2021) 184(8):2239–54.e39. doi: 10.1016/j.cell.2021.03.009 33831375 PMC8054914

[36] Jian M Nan L Guocheng J Qingfu Z Xueshan Q Enhua W. Downregulating prl-3 inhibit migration and invasion of lung cancer cell *Via* rhoa and Mdia1. Tumori (2012) 98(3):370–6. doi: 10.1700/1125.12407 22825514

[37] Lee J Kim H Lee JE Shin SJ Oh S Kwon G. Selective cytotoxicity of the nampt inhibitor Fk866 toward gastric cancer cells with markers of the epithelial-mesenchymal transition, due to loss of naprt. Gastroenterology (2018) 155(3):799–814 e13. doi: 10.1053/j.gastro.2018.05.024 29775598

[38] Fang H Niu K Mo D Zhu Y Tan Q Wei D. Recql4-aurora b kinase axis is essential for cellular proliferation, cell cycle progression, and mitotic integrity. Oncogenesis (2018) 7(9):68. doi: 10.1038/s41389-018-0080-4 30206236 PMC6134139

[39] Jiang W Xu J Liao Z Li G Zhang C Feng Y. Prognostic signature for lung adenocarcinoma patients based on cell-Cycle-Related genes. Front Cell Dev Biol (2021) 9:655950. doi: 10.3389/fcell.2021.655950 33869220 PMC8044954

