# Supplementary material for: Comprehensive analyses unveil novel genomic and immunological characteristics of micropapillary pattern in lung adenocarcinoma
Source: Front Oncol. 2022 Aug 3;12:931209. doi: 10.3389/fonc.2022.931209 (PMC9381833; doi:10.3389/fonc.2022.931209)

Suppl. Fig 1

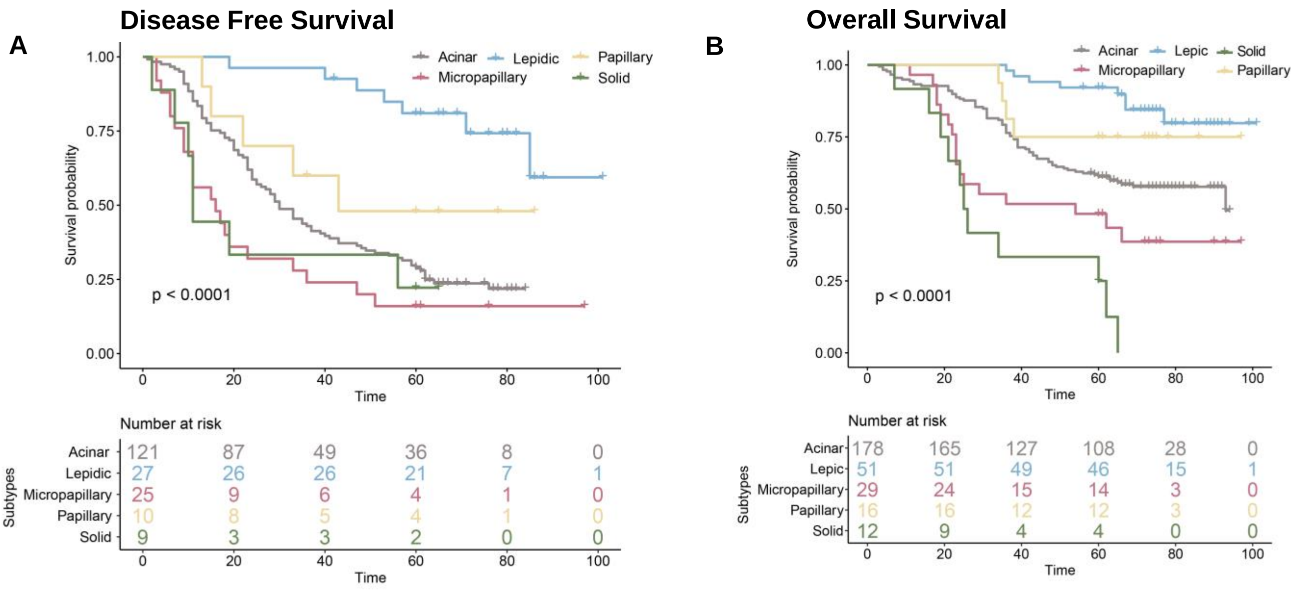
A
Disease Free Survival
Acinar
Lepidic
Papillary
Micropapillary
Solid
Survival probability
1.00
0.75
0.50
0.25
0.00
p < 0.0001
0 20 40 60 80 100
Time
Number at risk
Subtypes
Acinar 121 87 49 36 8 0
Lepidic 27 26 26 21 7 1
Micropapillary 25 9 6 4 1 0
Papillary 10 8 5 4 1 0
Solid 9 3 3 2 0 0
0 20 40 60 80 100
Time
B
Overall Survival
Acinar
Lepic
Solid
Micropapillary
Papillary
Survival probability
1.00
0.75
0.50
0.25
0.00
p < 0.0001
0 20 40 60 80 100
Time
Number at risk
Subtypes
Acinar 178 165 127 108 28 0
Lepic 51 51 49 46 15 1
Micropapillary 29 24 15 14 3 0
Papillary 16 16 12 12 3 0
Solid 12 9 4 4 0 0
0 20 40 60 80 100
Time

Suppl. Fig 2

A

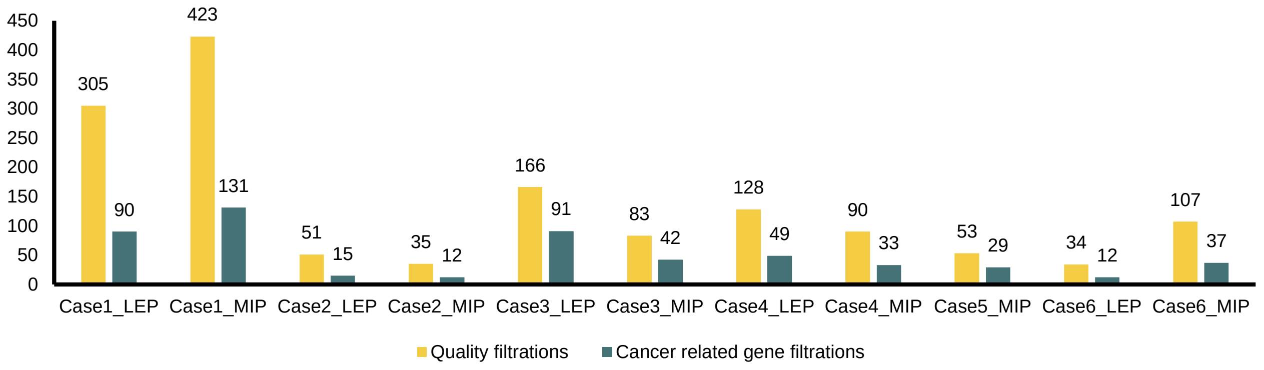

B

`	Lung-Broad			Lung-OncoSG			TCGA-LUAD		
	Number of samples								
	**LEP (n=13)**	**MIP (n=17)**	**Adj. P-value**	**LEP (n=10)**	**MIP (n=4)**	**Adj. P-value**	**LEP (n=12)**	**MIP (n=23)**	**Adj. P-value**
EGFR	4	2	1.49E-03	6	3	0.276613	2	4	0.000421
RYR2	1	7	0.787038	2	1	0.6564	2	7	0.734327
SI	1	5	0.559035	2	0	0.4025	1	4	0.251918
TP53	3	8	1	2	0	0.4025	4	8	1
TRIO	0	1	0.050863	0	0	0.117859	0	0	0.023253
XIRP2	2	3	0.415917	0	0	0.117859	3	5	0.48311
ABCC6	0	0	0.050863	0	0	0.117859	0	0	0.035716
BRWD3	1	2	0.454538	0	0	0.117859	0	2	0.123249
CEBPA	0	0	0.050863	0	0	0.117859	0	0	0.035716
COL22A1	1	2	0.454538	0	0	0.117859	1	1	0.123249
GTF3C1	2	1	0.454538	0	0	0.117859	1	1	0.123249
KALRN	1	2	0.454538	0	1	0.367744	0	0	0.035716
KCNT2	0	6	0.787038	1	1	0.651527	0	1	0.071703
LMBRD2	0	2	0.229164	0	0	0.117859	0	0	0.035716
NLGN1	1	1	0.229164	0	0	0.117859	0	3	0.198375
NLRP9	0	0	0.050863	0	0	0.117859	1	0	0.071703
PCLO	3	6	1	1	0	0.367744	0	5	0.43056
PDE4DIP	0	0	0.050863	0	0	0.117859	2	2	0.402863
PHRF1	0	1	0.133247	0	0	0.117859	0	1	0.071703
TTN	3	8	0.787038	0	2	0.651527	4	12	0.402863
UNC13C	0	5	0.787038	0	0	0.117859	0	1	0.071703
USP7	0	1	0.133247	0	1	0.367744	0	1	0.071703
XPO7	0	0	0.050863	0	0	0.117859	0	0	0.035716

C

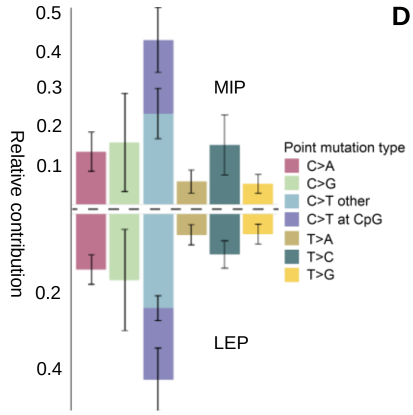

D

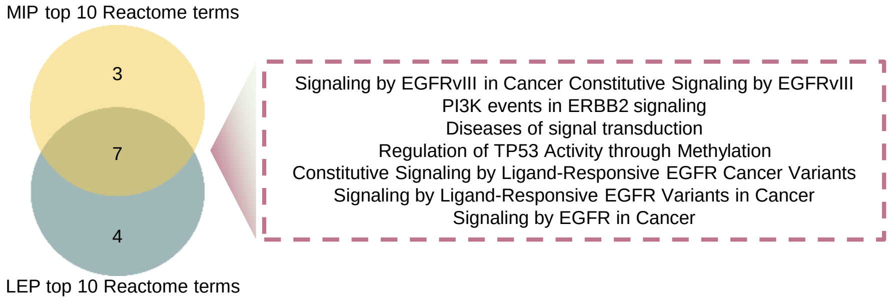

Suppl. Fig 3

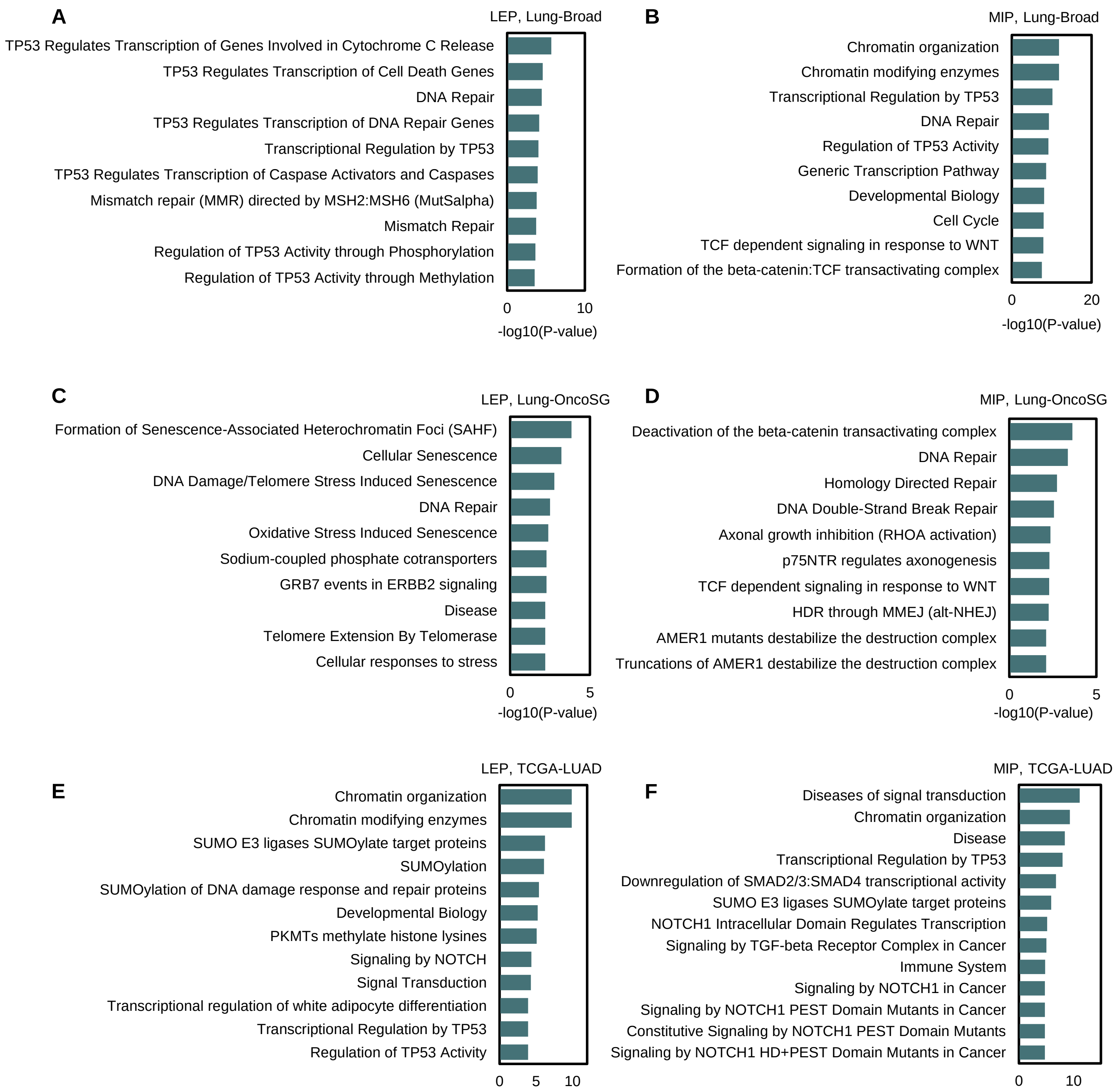
A
LEP, Lung-Broad
TP53 Regulates Transcription of Genes Involved in Cytochrome C Release
TP53 Regulates Transcription of Cell Death Genes
DNA Repair
TP53 Regulates Transcription of DNA Repair Genes
Transcriptional Regulation by TP53
TP53 Regulates Transcription of Caspase Activators and Caspases
Mismatch repair (MMR) directed by MSH2:MSH6 (MutSalpha)
Mismatch Repair
Regulation of TP53 Activity through Phosphorylation
Regulation of TP53 Activity through Methylation
0
10
-log10(P-value)
B
MIP, Lung-Broad
Chromatin organization
Chromatin modifying enzymes
Transcriptional Regulation by TP53
DNA Repair
Regulation of TP53 Activity
Generic Transcription Pathway
Developmental Biology
Cell Cycle
TCF dependent signaling in response to WNT
Formation of the beta-catenin:TCF transactivating complex
0
20
-log10(P-value)
C
LEP, Lung-OncoSG
Formation of Senescence-Associated Heterochromatin Foci (SAHF)
Cellular Senescence
DNA Damage/Telomere Stress Induced Senescence
DNA Repair
Oxidative Stress Induced Senescence
Sodium-coupled phosphate cotransporters
GRB7 events in ERBB2 signaling
Disease
Telomere Extension By Telomerase
Cellular responses to stress
0
5
-log10(P-value)
D
MIP, Lung-OncoSG
Deactivation of the beta-catenin transactivating complex
DNA Repair
Homology Directed Repair
DNA Double-Strand Break Repair
Axonal growth inhibition (RHOA activation)
p75NTR regulates axonogenesis
TCF dependent signaling in response to WNT
HDR through MMEJ (alt-NHEJ)
AMER1 mutants destabilize the destruction complex
Truncations of AMER1 destabilize the destruction complex
0
5
-log10(P-value)
E
LEP, TCGA-LUAD
Chromatin organization
Chromatin modifying enzymes
SUMO E3 ligases SUMOylate target proteins
SUMOylation
SUMOylation of DNA damage response and repair proteins
Developmental Biology
PKMTs methylate histone lysines
Signaling by NOTCH
Signal Transduction
Transcriptional regulation of white adipocyte differentiation
Transcriptional Regulation by TP53
Regulation of TP53 Activity
0
5
10
F
MIP, TCGA-LUAD
Diseases of signal transduction
Chromatin organization
Disease
Transcriptional Regulation by TP53
Downregulation of SMAD2/3:SMAD4 transcriptional activity
SUMO E3 ligases SUMOylate target proteins
NOTCH1 Intracellular Domain Regulates Transcription
Signaling by TGF-beta Receptor Complex in Cancer
Immune System
Signaling by NOTCH1 in Cancer
Signaling by NOTCH1 PEST Domain Mutants in Cancer
Constitutive Signaling by NOTCH1 PEST Domain Mutants
Signaling by NOTCH1 HD+PEST Domain Mutants in Cancer
0
10

Suppl. Fig 4

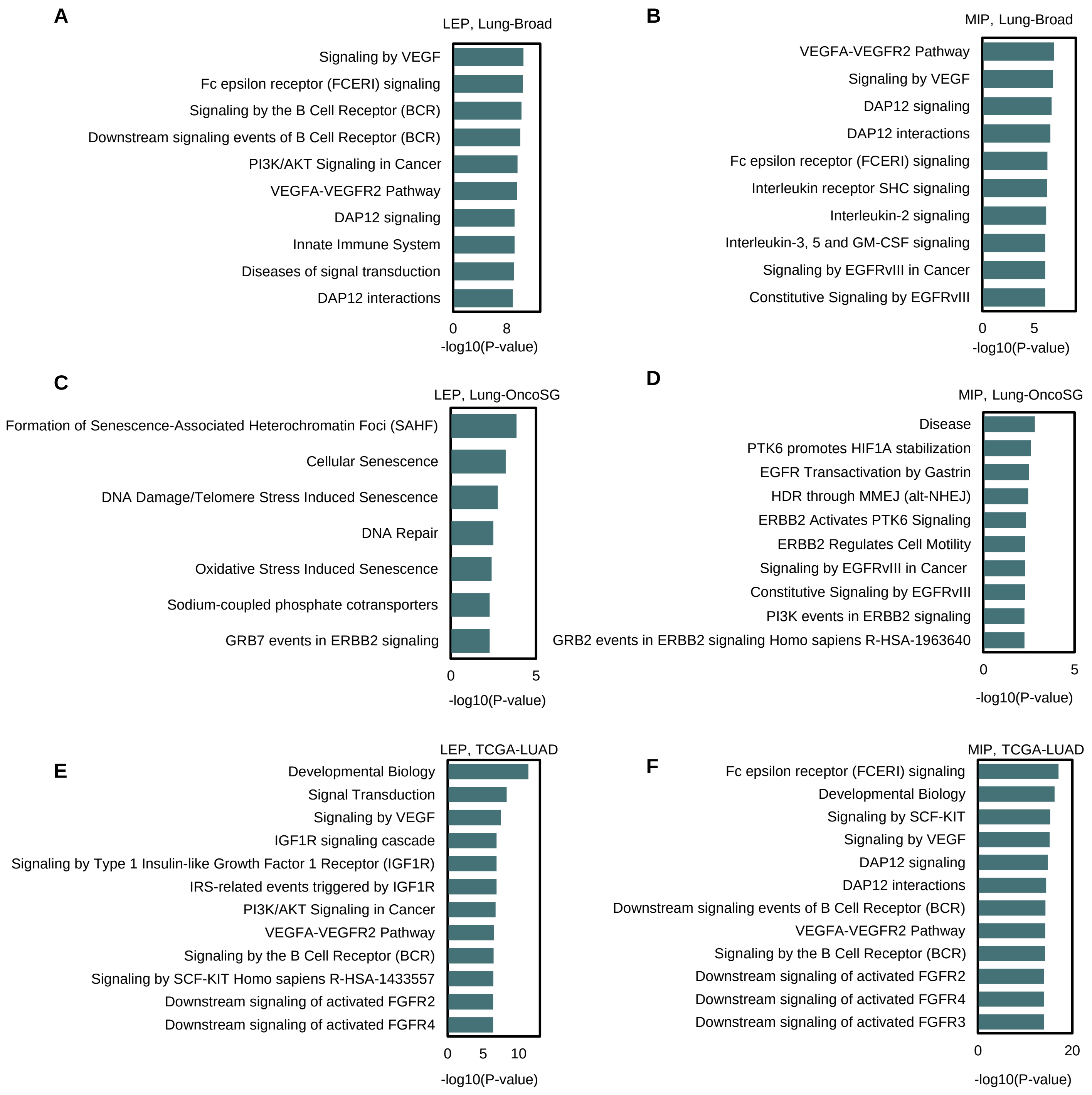
A
LEP, Lung-Broad
Signaling by VEGF
Fc epsilon receptor (FCERI) signaling
Signaling by the B Cell Receptor (BCR)
Downstream signaling events of B Cell Receptor (BCR)
PI3K/AKT Signaling in Cancer
VEGFA-VEGFR2 Pathway
DAP12 signaling
Innate Immune System
Diseases of signal transduction
DAP12 interactions
0
8
-log10(P-value)
B
MIP, Lung-Broad
VEGFA-VEGFR2 Pathway
Signaling by VEGF
DAP12 signaling
DAP12 interactions
Fc epsilon receptor (FCERI) signaling
Interleukin receptor SHC signaling
Interleukin-2 signaling
Interleukin-3, 5 and GM-CSF signaling
Signaling by EGFRvIII in Cancer
Constitutive Signaling by EGFRvIII
0
5
-log10(P-value)
C
LEP, Lung-OncoSG
Formation of Senescence-Associated Heterochromatin Foci (SAHF)
Cellular Senescence
DNA Damage/Telomere Stress Induced Senescence
DNA Repair
Oxidative Stress Induced Senescence
Sodium-coupled phosphate cotransporters
GRB7 events in ERBB2 signaling
0
5
-log10(P-value)
D
MIP, Lung-OncoSG
Disease
PTK6 promotes HIF1A stabilization
EGFR Transactivation by Gastrin
HDR through MMEJ (alt-NHEJ)
ERBB2 Activates PTK6 Signaling
ERBB2 Regulates Cell Motility
Signaling by EGFRvIII in Cancer
Constitutive Signaling by EGFRvIII
PI3K events in ERBB2 signaling
GRB2 events in ERBB2 signaling Homo sapiens R-HSA-1963640
0
5
-log10(P-value)
E
LEP, TCGA-LUAD
Developmental Biology
Signal Transduction
Signaling by VEGF
IGF1R signaling cascade
Signaling by Type 1 Insulin-like Growth Factor 1 Receptor (IGF1R)
IRS-related events triggered by IGF1R
PI3K/AKT Signaling in Cancer
VEGFA-VEGFR2 Pathway
Signaling by the B Cell Receptor (BCR)
Signaling by SCF-KIT Homo sapiens R-HSA-1433557
Downstream signaling of activated FGFR2
Downstream signaling of activated FGFR4
0
5
10
-log10(P-value)
F
MIP, TCGA-LUAD
Fc epsilon receptor (FCERI) signaling
Developmental Biology
Signaling by SCF-KIT
Signaling by VEGF
DAP12 signaling
DAP12 interactions
Downstream signaling events of B Cell Receptor (BCR)
VEGFA-VEGFR2 Pathway
Signaling by the B Cell Receptor (BCR)
Downstream signaling of activated FGFR2
Downstream signaling of activated FGFR4
Downstream signaling of activated FGFR3
0
20
-log10(P-value)

Suppl. Fig 5

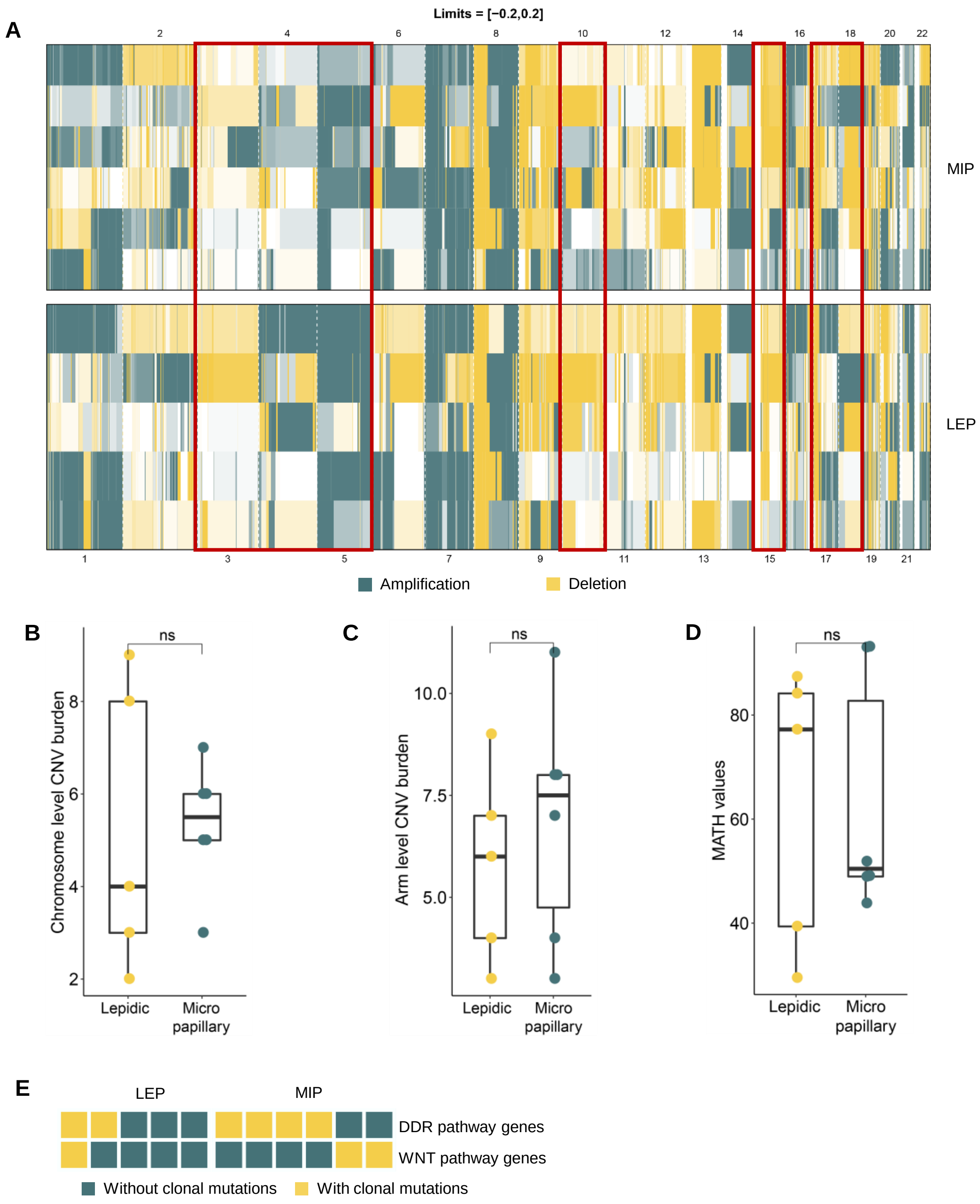

A
Limits = [−0.2,0.2]
2
4
6
8
10
12
14
16
18
20
22
MIP
LEP
1
3
5
7
9
11
13
15
17
19
21
Amplification
Deletion
B
ns
Chromosome level CNV burden
8
6
4
2
Lepidic
Micro papillary
C
ns
Arm level CNV burden
10.0
7.5
5.0
Lepidic
Micro papillary
D
ns
MATH values
80
60
40
Lepidic
Micro papillary
E
LEP
MIP
DDR pathway genes
WNT pathway genes
Without clonal mutations
With clonal mutations

Suppl. Fig 6

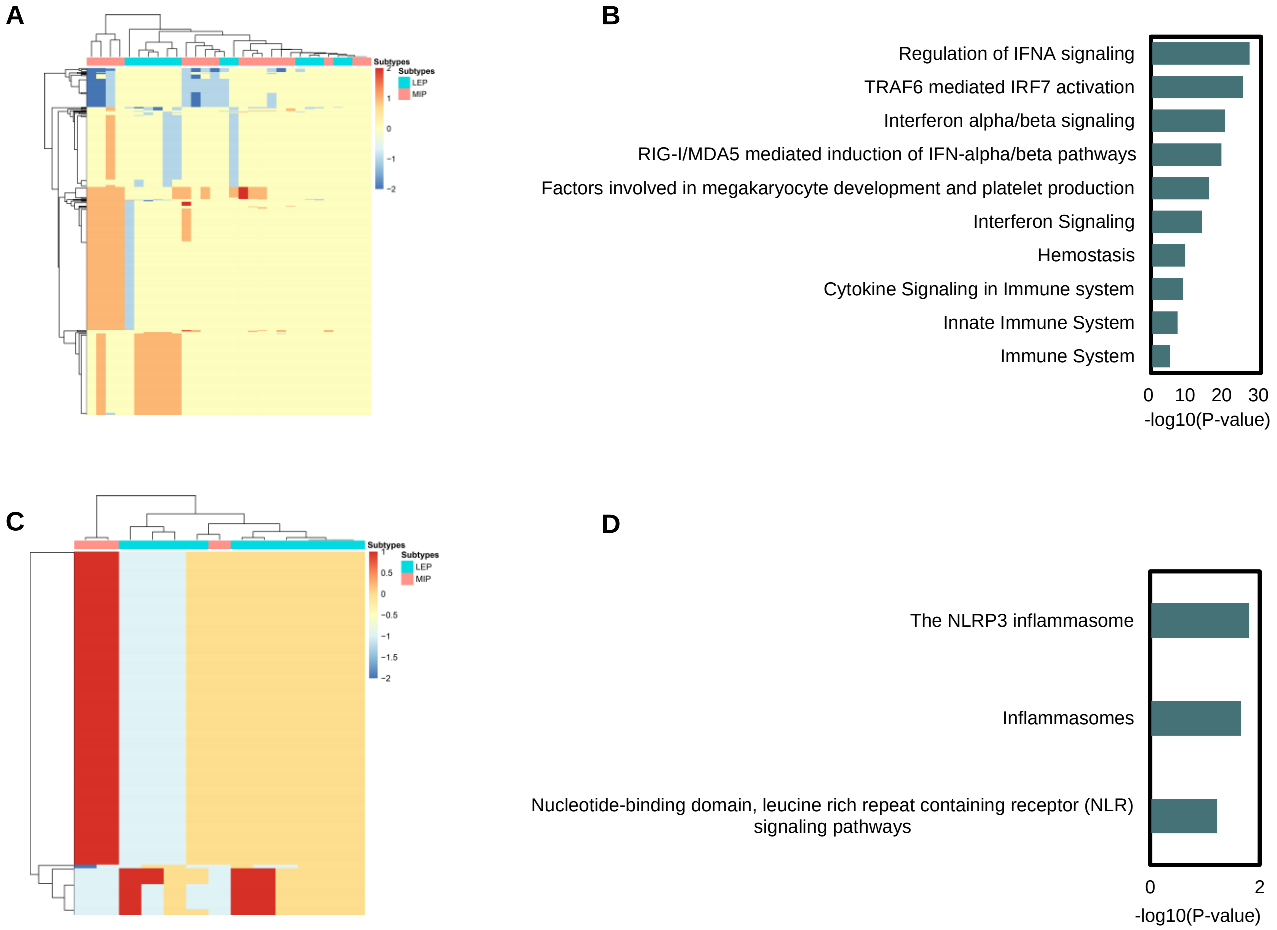
A
Subtypes
LEP
MIP
B
Regulation of IFNA signaling
TRAF6 mediated IRF7 activation
Interferon alpha/beta signaling
RIG-I/MDA5 mediated induction of IFN-alpha/beta pathways
Factors involved in megakaryocyte development and platelet production
Interferon Signaling
Hemostasis
Cytokine Signaling in Immune system
Innate Immune System
Immune System
0 10 20 30
-log10(P-value)
C
Subtypes
LEP
MIP
D
The NLRP3 inflammasome
Inflammasomes
Nucleotide-binding domain, leucine rich repeat containing receptor (NLR) signaling pathways
0 2
-log10(P-value)

Suppl. Fig 7

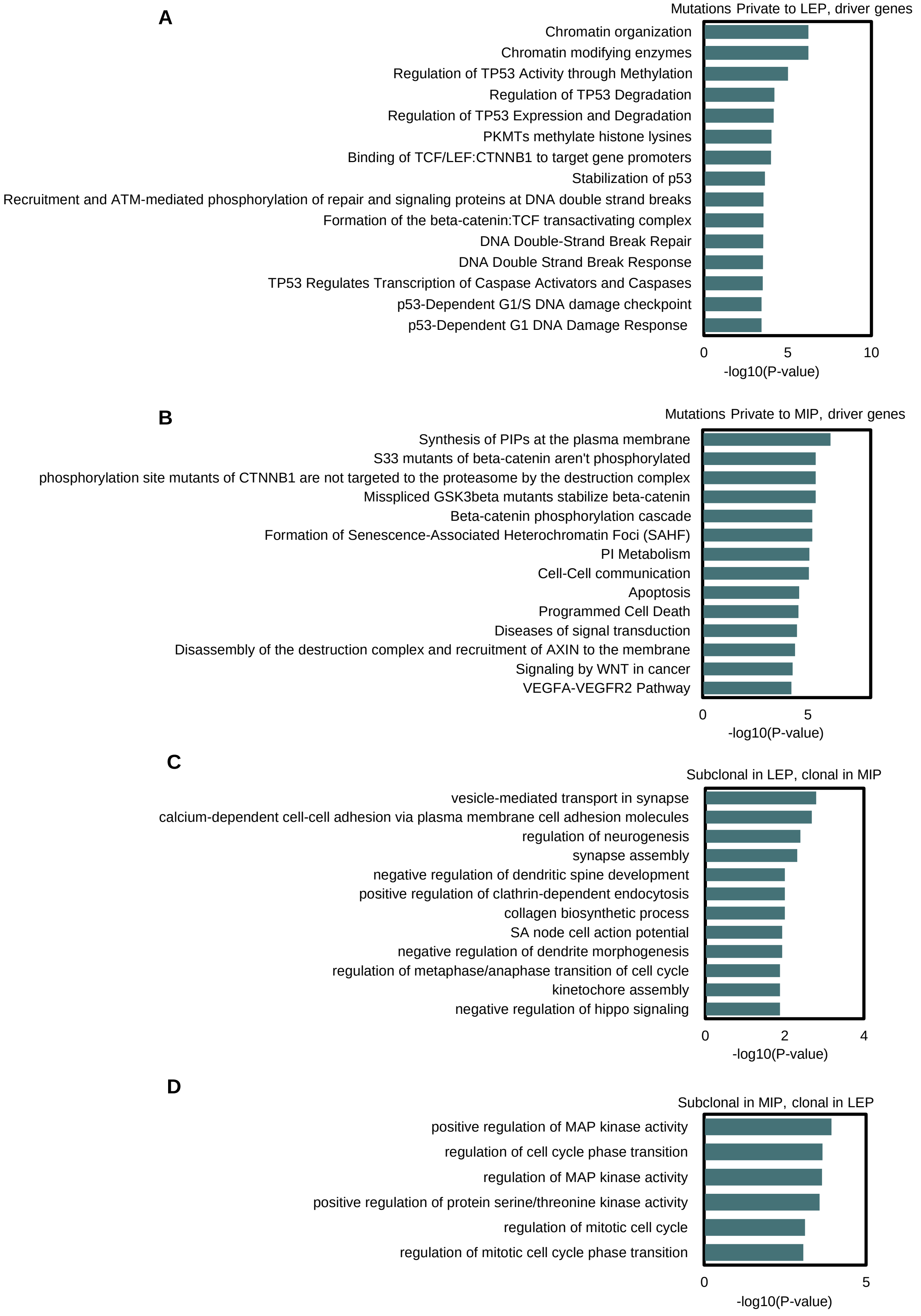
A
Mutations Private to LEP, driver genes
Chromatin organization
Chromatin modifying enzymes
Regulation of TP53 Activity through Methylation
Regulation of TP53 Degradation
Regulation of TP53 Expression and Degradation
PKMTs methylate histone lysines
Binding of TCF/LEF:CTNNB1 to target gene promoters
Stabilization of p53
Recruitment and ATM-mediated phosphorylation of repair and signaling proteins at DNA double strand breaks
Formation of the beta-catenin:TCF transactivating complex
DNA Double-Strand Break Repair
DNA Double Strand Break Response
TP53 Regulates Transcription of Caspase Activators and Caspases
p53-Dependent G1/S DNA damage checkpoint
p53-Dependent G1 DNA Damage Response
0
5
10
-log10(P-value)
B
Mutations Private to MIP, driver genes
Synthesis of PIPs at the plasma membrane
S33 mutants of beta-catenin aren't phosphorylated
phosphorylation site mutants of CTNNB1 are not targeted to the proteasome by the destruction complex
Misspliced GSK3beta mutants stabilize beta-catenin
Beta-catenin phosphorylation cascade
Formation of Senescence-Associated Heterochromatin Foci (SAHF)
PI Metabolism
Cell-Cell communication
Apoptosis
Programmed Cell Death
Diseases of signal transduction
Disassembly of the destruction complex and recruitment of AXIN to the membrane
Signaling by WNT in cancer
VEGFA-VEGFR2 Pathway
0
5
-log10(P-value)
C
Subclonal in LEP, clonal in MIP
vesicle-mediated transport in synapse
calcium-dependent cell-cell adhesion via plasma membrane cell adhesion molecules
regulation of neurogenesis
synapse assembly
negative regulation of dendritic spine development
positive regulation of clathrin-dependent endocytosis
collagen biosynthetic process
SA node cell action potential
negative regulation of dendrite morphogenesis
regulation of metaphase/anaphase transition of cell cycle
kinetochore assembly
negative regulation of hippo signaling
0
2
4
-log10(P-value)
D
Subclonal in MIP, clonal in LEP
positive regulation of MAP kinase activity
regulation of cell cycle phase transition
regulation of MAP kinase activity
positive regulation of protein serine/threonine kinase activity
regulation of mitotic cell cycle
regulation of mitotic cell cycle phase transition
0
5
-log10(P-value)

Suppl. Fig 8

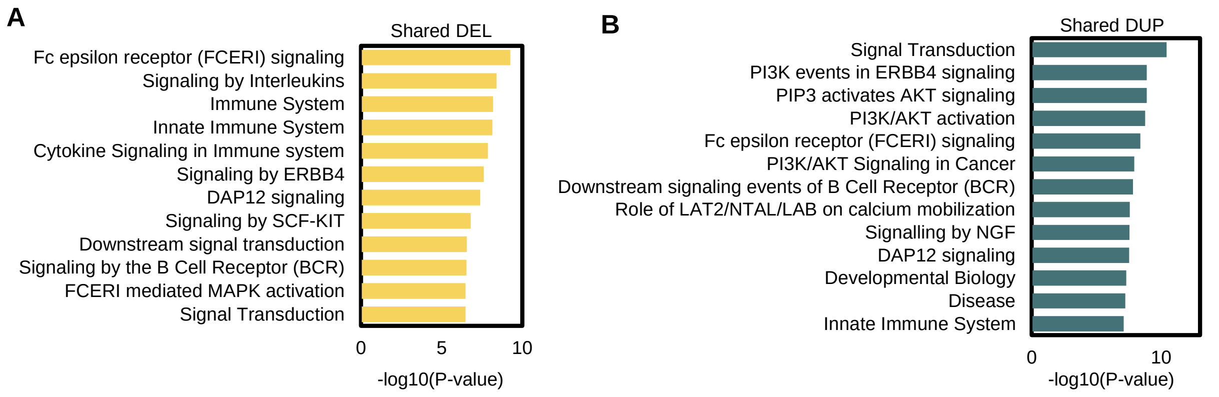
A
Shared DEL
Fc epsilon receptor (FCERI) signaling
Signaling by Interleukins
Immune System
Innate Immune System
Cytokine Signaling in Immune system
Signaling by ERBB4
DAP12 signaling
Signaling by SCF-KIT
Downstream signal transduction
Signaling by the B Cell Receptor (BCR)
FCERI mediated MAPK activation
Signal Transduction
0
5
10
-log10(P-value)
B
Shared DUP
Signal Transduction
PI3K events in ERBB4 signaling
PIP3 activates AKT signaling
PI3K/AKT activation
Fc epsilon receptor (FCERI) signaling
PI3K/AKT Signaling in Cancer
Downstream signaling events of B Cell Receptor (BCR)
Role of LAT2/NTAL/LAB on calcium mobilization
Signalling by NGF
DAP12 signaling
Developmental Biology
Disease
Innate Immune System
0
10
-log10(P-value)

Suppl. Fig 9

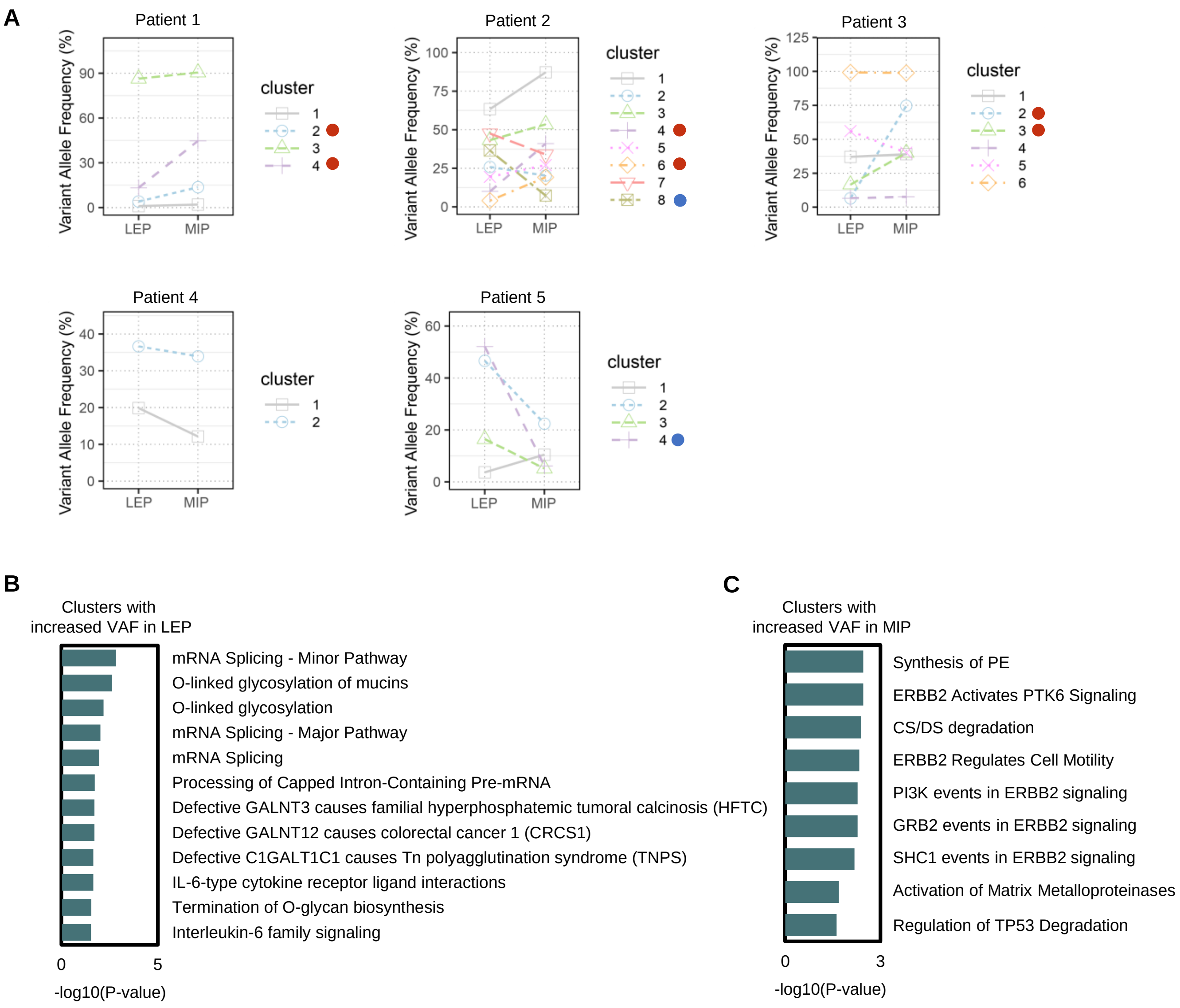
A
Patient 1
Variant Allele Frequency (%)
0
30
60
90
LEP
MIP
cluster
1
2
3
4
Patient 2
Variant Allele Frequency (%)
0
25
50
75
100
LEP
MIP
cluster
1
2
3
4
5
6
7
8
Patient 3
Variant Allele Frequency (%)
0
25
50
75
100
125
LEP
MIP
cluster
1
2
3
4
5
6
Patient 4
Variant Allele Frequency (%)
0
10
20
30
40
LEP
MIP
cluster
1
2
Patient 5
Variant Allele Frequency (%)
0
20
40
60
LEP
MIP
cluster
1
2
3
4
B
Clusters with increased VAF in LEP
mRNA Splicing - Minor Pathway
O-linked glycosylation of mucins
O-linked glycosylation
mRNA Splicing - Major Pathway
mRNA Splicing
Processing of Capped Intron-Containing Pre-mRNA
Defective GALNT3 causes familial hyperphosphatemic tumoral calcinosis (HFTC)
Defective GALNT12 causes colorectal cancer 1 (CRCS1)
Defective C1GALT1C1 causes Tn polyagglutination syndrome (TNPS)
IL-6-type cytokine receptor ligand interactions
Termination of O-glycan biosynthesis
Interleukin-6 family signaling
0
5
-log10(P-value)
C
Clusters with increased VAF in MIP
Synthesis of PE
ERBB2 Activates PTK6 Signaling
CS/DS degradation
ERBB2 Regulates Cell Motility
PI3K events in ERBB2 signaling
GRB2 events in ERBB2 signaling
SHC1 events in ERBB2 signaling
Activation of Matrix Metalloproteinases
Regulation of TP53 Degradation
0
3
-log10(P-value)

Suppl. Fig 10

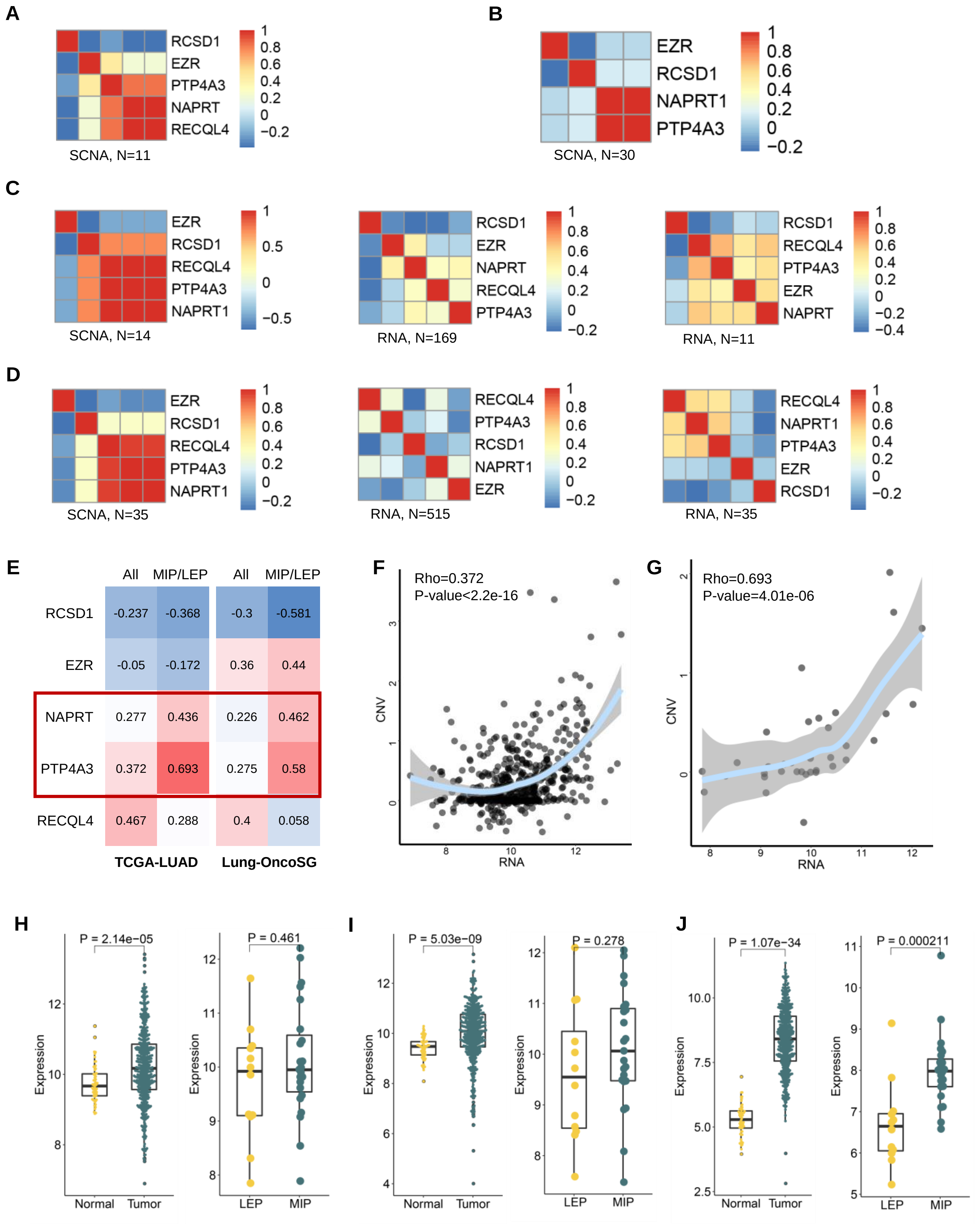

A
RCSD1
EZR
PTP4A3
NAPRT
RECQL4
SCNA, N=11
B
EZR
RCSD1
NAPRT1
PTP4A3
SCNA, N=30
C
SCNA, N=14
RNA, N=169
RNA, N=11
D
SCNA, N=35
RNA, N=515
RNA, N=35
E
All
MIP/LEP
RCSD1 -0.237 -0.368 -0.3 -0.581
EZR -0.05 -0.172 0.36 0.44
NAPRT 0.277 0.436 0.226 0.462
PTP4A3 0.372 0.693 0.275 0.58
RECQL4 0.467 0.288 0.4 0.058
TCGA-LUAD
Lung-OncoSG
F
Rho=0.372
P-value<2.2e-16
CNV
RNA
G
Rho=0.693
P-value=4.01e-06
CNV
RNA
H
P = 2.14e-05
P = 0.461
Expression
Normal
Tumor
LEP
MIP
I
P = 5.03e-09
P = 0.278
J
P = 1.07e-34
P = 0.000211

Suppl. Fig 11

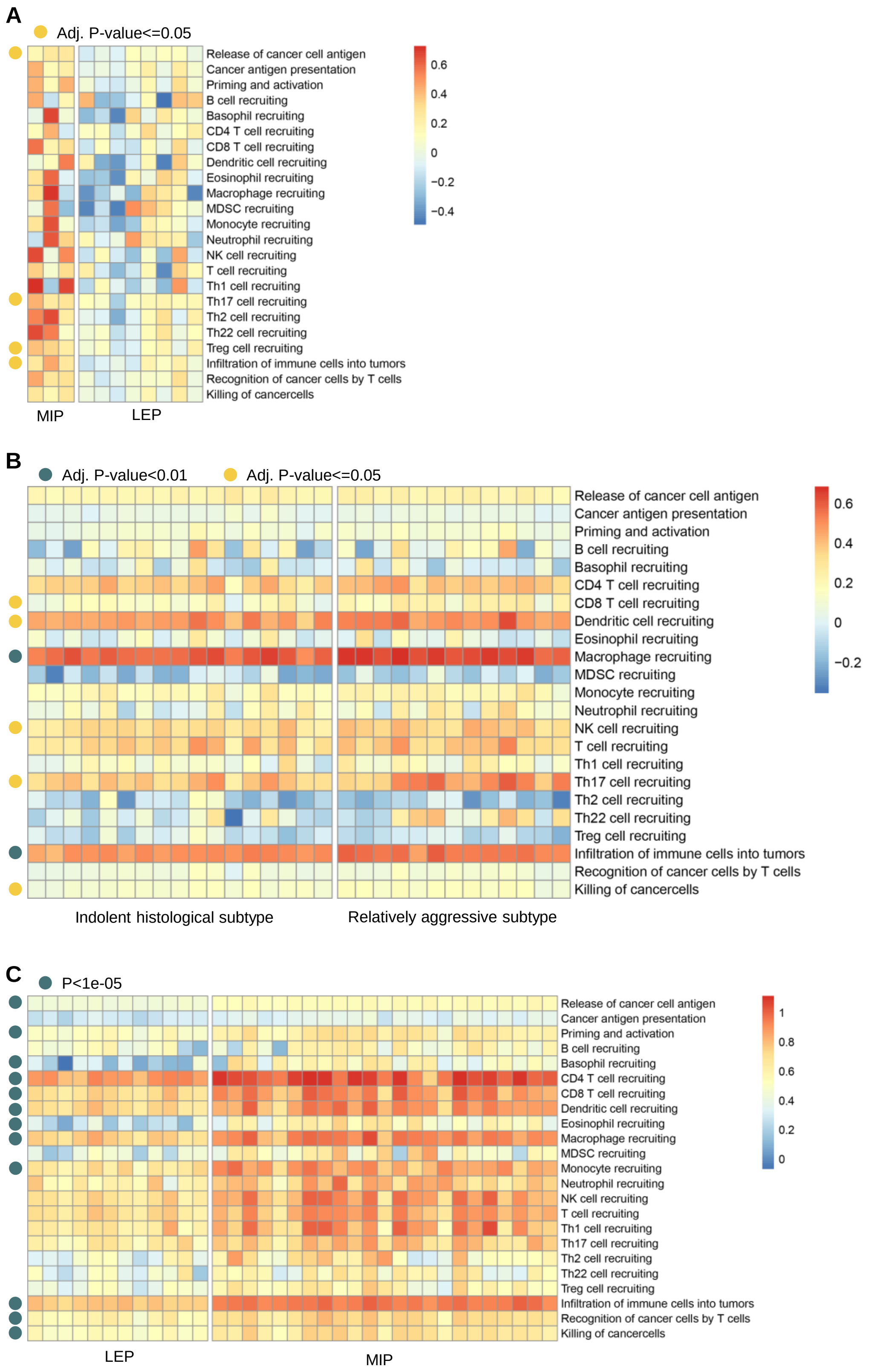
A
Adj. P-value<=0.05
Release of cancer cell antigen
Cancer antigen presentation
Priming and activation
B cell recruiting
Basophil recruiting
CD4 T cell recruiting
CD8 T cell recruiting
Dendritic cell recruiting
Eosinophil recruiting
Macrophage recruiting
MDSC recruiting
Monocyte recruiting
Neutrophil recruiting
NK cell recruiting
T cell recruiting
Th1 cell recruiting
Th17 cell recruiting
Th2 cell recruiting
Th22 cell recruiting
Treg cell recruiting
Infiltration of immune cells into tumors
Recognition of cancer cells by T cells
Killing of cancercells
0.6
0.4
0.2
0
−0.2
−0.4
MIP
LEP
B
Adj. P-value<0.01
Adj. P-value<=0.05
Release of cancer cell antigen
Cancer antigen presentation
Priming and activation
B cell recruiting
Basophil recruiting
CD4 T cell recruiting
CD8 T cell recruiting
Dendritic cell recruiting
Eosinophil recruiting
Macrophage recruiting
MDSC recruiting
Monocyte recruiting
Neutrophil recruiting
NK cell recruiting
T cell recruiting
Th1 cell recruiting
Th17 cell recruiting
Th2 cell recruiting
Th22 cell recruiting
Treg cell recruiting
Infiltration of immune cells into tumors
Recognition of cancer cells by T cells
Killing of cancercells
0.6
0.4
0.2
0
−0.2
Indolent histological subtype
Relatively aggressive subtype
C
P<1e-05
Release of cancer cell antigen
Cancer antigen presentation
Priming and activation
B cell recruiting
Basophil recruiting
CD4 T cell recruiting
CD8 T cell recruiting
Dendritic cell recruiting
Eosinophil recruiting
Macrophage recruiting
MDSC recruiting
Monocyte recruiting
Neutrophil recruiting
NK cell recruiting
T cell recruiting
Th1 cell recruiting
Th17 cell recruiting
Th2 cell recruiting
Th22 cell recruiting
Treg cell recruiting
Infiltration of immune cells into tumors
Recognition of cancer cells by T cells
Killing of cancercells
1
0.8
0.6
0.4
0.2
0
LEP
MIP

Suppl. Fig 12

A

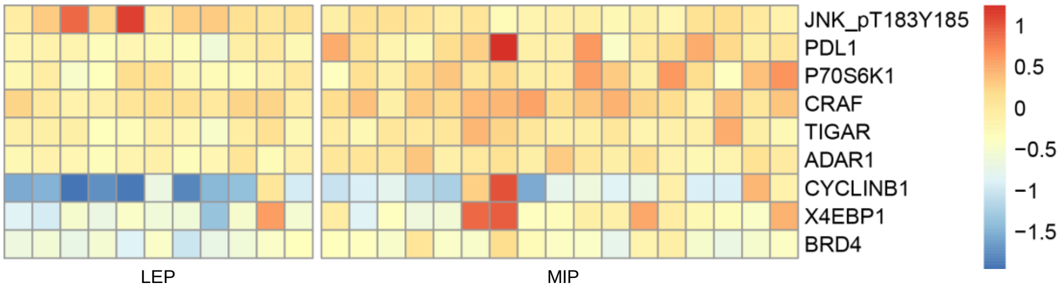

B

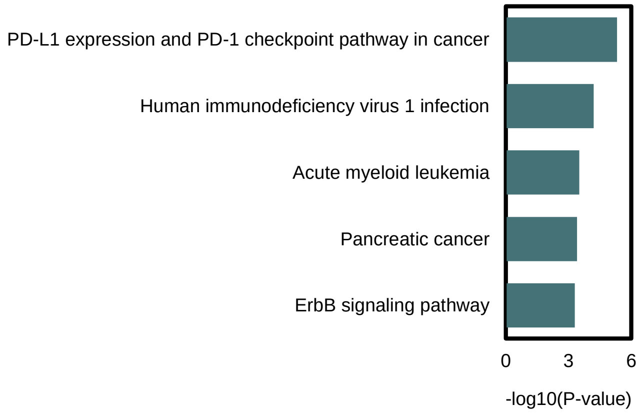

Supplement: Supplementary file 1 [file Presentation_1.pdf]
